# Variation in primary metabolites in parental and near-isogenic lines of the QTL *qDTY*_*12.1*_: altered roots and flag leaves but similar spikelets of rice under drought

**DOI:** 10.1007/s11032-015-0322-5

**Published:** 2015-06-02

**Authors:** Manish L. Raorane, Isaiah M. Pabuayon, Berta Miro, Rajesh Kalladan, Mohammad Reza-Hajirezai, Rowena H. Oane, Arvind Kumar, Nese Sreenivasulu, Amelia Henry, Ajay Kohli

**Affiliations:** International Rice Research Institute (IRRI), DAPO 7777, Metro Manila, Philippines; Leibniz Institute of Plant Genetics and Crop Plant Research (IPK), Corrensstrasse 03, 06466 Gatersleben, Germany; Institute of Plant and Microbial Biology, Academia Sinica, 128 Sec. 2, Academia Road, Nankang, Taipei 11529 Taiwan

**Keywords:** Drought, Carbon, Nitrogen, Remobilization, Root, Flag leaf, QTL, Grain yield, Metabolomics, Rice

## Abstract

There is a widespread consensus that drought will mostly affect present and future agriculture negatively. Generating drought-tolerant crops is thus a high priority. However complicated the underlying genetic and regulatory networks for differences in plant performance under stress are, they would be reflected in straightforward differences in primary metabolites. This is because primary metabolites such as amino acids and sugars form the building blocks of all pathways and processes for growth, development, reproduction, and environmental responses. Comparison of such differences was undertaken between the parental line and a near-isogenic line of *qDTY*_*12.1*_, a QTL for rice yield under drought. The comparison was informative regarding the effect of the QTL in three genetic backgrounds: donor, recipient, and improved recipient, thus illustrating the gene × gene (G × G) interactions. Such a comparison when extended to well-watered and drought conditions illustrated the gene × environment (G × E) interactions. Assessment of such G × G and G × E responses in roots, flag leaves, and spikelets added a yet more informative dimension of tissue-specific responses to drought, mediated by *qDTY*_*12.1*_. Data on variation in primary metabolites subjected to ANOVA, Tukey’s test, Welch’s *t* test, and PCA underscored the importance of the roots and demonstrated concordance between variation in metabolites and morpho-physiological responses to drought. Results suggested that for gainful insights into rice yield under drought, rather than vegetative stage drought tolerance, multiple tissues and genotypes must be assessed at the reproductive stage to avoid misleading conclusions about using particular metabolites or related genes and proteins as candidates or markers for drought tolerance.

**Electronic supplementary material:**

The online version of this article (doi:10.1007/s11032-015-0322-5) contains supplementary material, which is available to authorized users.

## Introduction

Plants respond and adapt to stress by making essential alterations across all functional levels, from molecules to whole-plant physiology (Hasanuzzaman et al. 2013). Over the last few decades, substantial research has gone into elucidating plant stress responses at all such levels. Under the present and predicted climate change scenarios, the possibility of climate-resilient agriculture through crop diversification is an increasingly supported concept (Lin 2011). However, generating drought-tolerant crops is of high priority because drought is the most indiscriminate and widespread abiotic stress. It is predicted to become increasingly destructive to agriculture and to agrarian society in the future (Grayson 2013).

Rice is one of the most important food crops and a staple for more than 50 % of the world’s population. As an important staple, rice has a central position in global food security and is directly related to alleviating hunger and poverty in Asia and Africa. Rice productivity is affected by adverse environmental conditions (Jagadish et al. 2012), and the reproductive stage water stress is particularly debilitating (O’Toole 1982). Global rice production suffers considerably due to drought (Serraj et al. 2011).

For generating drought-tolerant rice, the three options are conventional, marker-assisted, or transgenic breeding approaches. Conventional breeding has been successful but temporally demanding, while the latter two approaches offer advantages of modern technologies to fast-track breeding. However, despite increasing precision in genome engineering, the transgenic approach is lumbered with inhibitory costs and refractory philosophies. Additionally, after nearly three decades of investments, while the technologies have exponentially improved, QTL and transgenic research-mediated success has been limited, and no rice genotype more tolerant than the landraces could be generated for commercial acceptance. This is in part due to the lack of a deeper and biologically relevant understanding of the omics data for molecular mechanisms involved in drought tolerance. Deciphering such mechanisms is challenging due to the inherently intricate relationships between genes, transcripts, proteins, and metabolites and also because of the panoply of mechanisms operative in rice under the various eco-geographies that it inhabits (Fukai and Cooper 1995; Swamy et al. 2011). For example, four major ecosystems for rice are generally recognized: irrigated rice, rainfed lowland, rainfed upland, and flood-prone areas (50, 34, 9, and 7 %, respectively, of the total area under rice; Khush 1997). The main difference between the two rainfed ecosystems is generally the lack of flooding problems in the upland ecosystem (Mackill et al. 1996). Given that rain is the primary source of water in rainfed ecosystem, drought has a particularly negative effect on upland areas in which the water drains down, causing drought conditions to be even more debilitating (Khush 1997).

Rice of the *indica* sub-type is found mainly in the lowlands of Asian tropics. Along with *japonica*, it is considered one of the two main genetic sub-types of rice. The early-maturing *indica* varieties known as *aus*, generally grown during the summer months of March to June due to their drought tolerance (Garris et al. 2005), have been shown by genome sequence comparisons to be genetically more distant than previously described (Schatz et al. 2014). This genetic variation includes genes for stress tolerance in *aus* varieties, increasing the importance of this subgroup to plant breeders for developing stress-tolerant lines. Several genes, morpho-physiological traits, breeding lines, and QTLs for drought tolerance have been identified in rice (Fukai and Cooper 1995; Swamy et al. 2011; Mir et al. 2012). Their validity is mostly at the vegetative stage, with no effect on yield under stress. Also, their inconsistency under different environments and in different genetic backgrounds has restricted the success in generating widespread drought-tolerant rice. Nevertheless, in the recent past, screening for yield under drought stress has led to promising success. The QTL *qDTY*_*12.1*_ for consistent large effects on grain yield under reproductive stage drought stress was identified on chromosome 12 in the population generated from crossing an *aus* and an *indica* rice genotype, i.e., Vandana and Way Rarem, respectively (Bernier et al. 2007). Vandana is able to produce yield under drought conditions, particularly in Indian upland ecosystems. Way Rarem is an Indonesian rice variety very susceptible to drought at reproductive stage, which is otherwise high yielding.

To date, this *qDTY*_*12.1*_ exhibits the largest effect on grain yield under drought in multiple genetic backgrounds, in diverse rice ecosystems, and at different growth stages of the rice plant (Bernier et al. 2009a; Mishra et al. 2013; Henry et al. 2014). Earlier studies dissecting the physiological basis of *qDTY*_*12.1*_ function indicated that grain yield under drought was improved mainly through increased root water uptake under water-limited conditions (Bernier et al. 2009b). Various near-isogenic lines (NILs) for *qDTY*_*12.1*_ were generated in the Vandana background. Field trials of these NILs indicated 30–40 % additive effect on yield under drought conditions (Henry et al. 2014). One NIL that consistently surpassed the tolerant parent Vandana for yield under drought was 481-B, whose genotype is identical to Vandana except for *qDTY*_*12.1*_ introgressed from Way Rarem (Kumar et al. 2014). Comparing two such genetically similar lines, i.e., the wild-type recipient Vandana and the NIL 481-B evidently improved for yield under drought, could provide an insight into the mechanisms underlying the morpho-physiological differences leading to yield. This would be especially useful since 481-B is valid in multi-environment field tests (Bernier et al. 2009a; Henry et al. 2014).

We chose to make such a comparison for targeted primary metabolites in roots, flag leaves, and spikelets of the parental genotypes Vandana and Way Rarem and the well-performing NIL 481-B under well-watered and drought conditions. The NIL 481-B exhibited 94.6 % recovery of the Vandana genome in the BC_2_F_4_ generation. Primary metabolites such as amino acids, sugars, starch, total carbon and nitrogen contents have intrinsic, constitutive functions in physiological processes and are directly involved in responding to internal stimuli of growth, development, and reproduction or to external biotic and abiotic environmental stimuli. This study centered on the hypothesis that comparatively better survival and grain yield of 481-B under drought must be reflected in tissue- and genotype-specific differences in such primary metabolites, however complicated the underlying genetic or regulatory networks invoked by the genes that underpin *qDTY*_*12.1*_ functionality are.

In comparison with regular “omics” studies between two highly contrasting genotypes for a trait, four features of our study make the results rather unique, i.e., (1) QTL *qDTY*_*12.1*_ comes from the susceptible genotype Way Rarem and not from the tolerant Vandana (Bernier et al. 2007). Therefore, NIL 481-B in the Vandana background allowed comparison of a tolerant genotype to its even more tolerant NIL. (2) Since the phenology of Vandana and 481-B was not changed, the sampling process captured the same growth stage and both, the source (roots and flag leaves) and sink (spikelets) tissue of a susceptible, tolerant, and improved genotype. (3) Sampling of the tissue was done under field conditions to represent on-farm situations. (4) NIL 481-B has an established field value for yield under drought according to multi-genotype and multi-environment field testing (Henry et al. 2014). The experimental material thus had an overarching value to the results obtained. Similarly, the experimental setup was also rigorous; multiple biological and technical replicates and rigorous statistical treatment of such inter-tissue and inter-genotype comparisons further confirmed the contributions of *qDTY*_*12.1*_ in increasing rice yield under drought.

For all practical purposes, reference to drought tolerance of genotypes used in this study implies yield under drought. Rice plants were grown in upland field conditions and exposed to drought at the most crucial phase of reproductive stage stress (O’Toole 1982), i.e., starting from panicle booting until the start of grain filling. Transcript expression level was also assessed for particular genes related to the primary metabolites that showed differential responses.

Even in its limited scope for the number of metabolites analyzed, our assessment highlighted the composite nature of drought tolerance and reiterated the G × G and G × E interactions necessary for the QTL to be useful. Important insights emerged from variations in the content of particular metabolites in different tissues, for their relevance to grain yield under drought. There were variations in the content of a larger number of metabolites in roots than in the flag leaves and spikelets. Overall, primary metabolite content variations supported the observed morpho-physiological changes in increased root and panicle branching and leaf water use efficiency associated with the success of the NILs for grain yield under drought. Although more extensive omics and gene function studies, some originating as a consequence of this study, are still required, important conclusions could be drawn from the present set of results. The versatile and farmers’ field tested NIL 481-B emerged as a useful model to gain further insights into the molecular networks operative for yield under drought. The relevance of our study and its results lies in the relationship between Vandana and its NIL 481-B, whereby the latter is similar to Vandana, except for the *qDTY*_*12.1*_ region. Changes in metabolites between the lines may thus be directly correlated with effects of *qDTY*_*12.1*_ or its interaction with other genes in Vandana. The latter effects could be dependably correlated due to data available for the response of Way Rarem as well. Important conclusions from our study were that *qDTY*_*12.1*_ affected the metabolic pathways in the tissues rather than the tissue itself. Comparatively, greater changes in roots and flag leaves most likely underlie the relatively unchanged characters of the spikelets. The altered metabolic pathways were a consequence of G × E and G × G interactions rather than of a single dominant gene effect of the donating parent.

## Materials and methods

### Plant material

Seeds of the rice cultivars Vandana and Way Rarem, which are the two parental genotypes of a cross used in identifying QTL *qDTY*_*12.1*_ for yield under drought (Bernier et al. 2007), as well as the seeds of the best performing NIL 481-B (Kumar et al. 2014; Henry et al. 2014), were used in this analysis. All seeds were from the International Rice Research Institute (IRRI), Philippines. To break seed dormancy, seeds were incubated at 50 °C for 5 days.

### Experimental design

Experiments were conducted both in vitro and in the field. The in vitro experiment was conducted in a controlled room (30 °C, room humidity, 24-h day cycle, 100 µmol m^−2^ s PAR). Twenty seeds of each genotype were dehulled, sterilized following standard procedures with ethanol and sodium hypochlorite, thoroughly rinsed, and dried. Each seed was then sown in sterile MS media for 4 days and then transplanted to Murashige and Skoog (MS) media with 10 % PEG-8000 for the simulated drought-stress treatment. Control plants were kept in MS throughout the experiment. Plants were collected for root morphology analyses at 14 days after sowing (DAS).

The field experiment was conducted at IRRI (Los Baños, Laguna, 14°10′11.81″N, 121°15′39.22″E) during the 2012 dry season. Seeds of Vandana, Way Rarem, and 481-B were sown into rotovated soil at a rate of 2.0 g m^−1^ into plots of 3 rows × 3 m. The three genotypes were sown in three replications in a randomized complete block design. The late-flowering Way Rarem was sown 20 days before the other genotypes in order to synchronize drought stress with flowering stage. Soil properties of this experiment were described in Henry et al. (2014). Fertilizer and field management followed standard agronomical practices at IRRI. Two water treatments were followed: a well-watered (WW) treatment in which sprinkler irrigation was applied three times per week throughout the study and a drought-stress (DS) treatment in an automated field rainout shelter in which irrigation was stopped at 35 days after sowing. Soil water potential in the DS treatment was monitored by tensiometers (Soilmoisture Equipment Corp., CA, USA; one per replicate) installed at a depth of 30 cm. From the date that Way Rarem plots were sown until harvest, the ambient temperature averaged 23.4–30.8 °C (min–max), relative humidity averaged 85.8 %, the crop received 1750 MJ m^−2^ solar radiation, and pan evaporation totaled 552 mm. Sampling was done at about 10 days after fertilization.

### Measurement of photosynthesis and stomatal conductance

For measurements of photosynthesis and stomatal conductance, a LI-6400 portable gas exchange system (Li-Cor Inc., Lincoln, NE) was used. Experiments were performed during the 2012 wet season. However, soil moisture status was similar in the drought treatments of different seasons. Light-response curves were conducted for Vandana and 481-B in the drought-stress and well-watered treatments. CO_2_-response curves were also conducted on these two treatments and genotypes. Settings for all measurements were based on ambient conditions and included a leaf temperature of 30 °C and a flow rate to maintain relative humidity at 65 %. The CO_2_ level was set to 400 ppm for the light-response curves, and the light level was set to 1000 μmol m^−2^ s^−1^ for the CO_2_ response curves. All measurements were replicated three times.

### Root and panicle morphology analysis

At 14 DAS, five seedlings of the control and PEG treatments were carefully pulled from the tubes, and the roots were washed in deionized water. Intact roots were then placed in a transparent tray filled with deionized water, scanned to acquired images at a resolution of 300 dpi (Epson Perfection V700 PHOTO), and an output was generated as a 256 grayscale TIFF file. Photographs of fully matured panicles from soil grown plants were taken using a digital SLR camera (Nikon D90).

### Sample preparation for sugar and metabolite analyses

Plant material for metabolite and sugar analysis was prepared before each specific procedure, which are described separately below. Tissue samples from developing spikelets, flag leaves, stems, and root crowns from three different plants of each genotype were collected from the field study. Tissue samples for all genotypes were collected at 71 DAS (91 DAS for Way Rarem). After collection, all samples were wrapped in aluminum foil and placed directly into liquid nitrogen before storing at −80 °C until further analysis. The plant material was lyophilized and then ground to a fine powder with liquid nitrogen prior to analysis. Each different tissue was processed separately, and each sample was analyzed in triplicate.

### Estimation of starch concentration

Starch was estimated by measuring glucose generated through enzymatic hydrolysis of starch. Amyloglucosidase (EC 3.2.1.3) breaks down starch in the presence of water, liberating single glucose sugars. Glucose is phosphorylated by ATP in the reaction catalyzed by hexokinase to form glucose-6-phosphate. Glucose amounts are thus estimated by measuring the NADH absorption at 340 nm which was generated during the conversion of glucose-6-phosphate to 6-phosphogluconate by the enzyme glucose-6-phosphate dehydrogenase (EC 1.1.1.49). The pellet obtained (from 15 to 20 mg of seed or leaf) after ethanolic extraction was used for starch estimation by HCl. The pellet was dissolved in 2 N HCl (1.5 mL) and incubated at 95 °C for 1 h. The resulting mixture was directly used for glucose estimation after centrifugation at 13,000*g* for 5 min. Briefly, a mixture of 750 μL of 100 mM imidazole buffer (pH 6.9) consisting of 2 mM NAD and 1 mM ATP was incubated at room temperature for 10 min in a disposable plastic cuvette along with 5–10 μl of the extract and 2 μL of glucose-6-phosphate dehydrogenase (2 units). After recording the initial absorbance of the mixture at 340 nm, 10 μL of hexokinase (EC 2.7.1.1, 8 units) solution was added to the mixture and incubated for an additional 25 min, and the absorbance at 340 nm was recorded. A standard curve was prepared using starch standards from maize kernels.

### Estimation of sugar concentration

Lyophilized powdered tissue sample (100 mg) was extracted thrice with 80 % v/v ethanol. The supernatant obtained after centrifugation was then evaporated to dryness using a vacuum centrifuge evaporator. The dried material was re-dissolved in deionized water, vortexed thoroughly, and filtered (Ultrafree-MC Membranes; Millipore) prior to sugar analysis by HPAEC.

Soluble sugars were analyzed by ion chromatography, HPAEC-PAD (high-performance anion-exchange chromatography–pulsed amperometric detection). Chromatographic analysis was conducted with a Dionex IC system consisting of a GP 50 gradient pump with an AS 50 auto-sampler, an ED40 electrochemical detector with setup shift and Ag/AgCl reference potential. Data acquisition and processing were accomplished with Dionex Chromeleon 6.70 software. Chromatographic separation was carried out with the analytical column CarboPac PA 20 in conjunction with a guard column and an Ion Pac trap guard column (Dionex Corporation, Oakville, Canada).

Column temperature was maintained at 35 °C in a column oven (STH-585). Analytes were separated with isocratic elution using 50 % 150 mM NaOH and 50 % water as eluent at a flow rate of 0.3 mL min^−1^ for 15 min. Analyte detection was achieved by applying a quadrupole-potential waveform (E1 = 0.1 V from 0 to 0.4 ms; E2 = 2.0 V from 0.41 to 0.42 ms; E3 = 0.6 V from 0.42 to 0.43 ms; E4 = –0.1 V from 0.4 to 0.5 ms). The analytical data quality was controlled by standard addition methods.

### Estimation of amino acids by HPLC

Lyophilized powdered tissue sample was extracted thrice with 80 % v/v ethanol, evaporated to dryness, re-dissolved in deionized water, and filtered prior to estimation of amino acids (as described in the sugars section). Derivatization was carried out according to the instructions provided in the manual, AccQ-Tag method (Meyer et al. 2008). Before the chromatographic analysis, the system was equilibrated with 100 % eluent A (140 mM sodium acetate and 7 mM triethanolamine) at 37 °C, and the fluorescence detector was set at 248 nm wavelength for excitation and 395 nm for absorbance.

The HPLC system consisted of a P680 pump with ASI-100 auto-sampler, an AccQ-Tag analytic column coupled to a Nova-Pak C18 guard column (Waters Corporation, Milford, MA) and an RF2000 fluorescent detector, managed by Chromeleon software version 6.70 (Dionex Corporation, Oakville, Canada). The mobile phase was delivered at a flow rate of 1 mL min^−1^ and maintained at 37 °C. From 0 to 50 min, the concentration of B (Acetonitrile) was raised linearly to 0 % A, 60 % B, and 40 % water, providing the gradient. Reagents were purchased from Waters Corp. (Milford, MA). The between-run precision error was minimized by programming random access to samples, standards, and replicates—all controlled externally through Chromeleon.

The different free amino acid derivatives were identified on the basis of their retention time as compared to the fluorescent response of the peak of each corresponding standard (amino acid standard, Waters Corporation, Milford, MA; Asp and Glu standards purchased separately, Sigma, Oakville, Canada).

Colorimetric assay of free proline content was conducted using the ninhydrin assay (Bates et al. 1973).

### Analysis of carbon and nitrogen contents

Carbon and nitrogen contents were analyzed using the Dumas’ combustion principle with a Vario EL *III* element analyzer (Elementar Analysensysteme GmbH, Hanau, Germany) in the CN operation mode. About 3–4 mg of oven-dried sample was weighed in an aluminum capsule, folded, and placed in the auto-sampler. Empty aluminum capsules were included as blank samples. The instrument was previously calibrated with sulfanic acid (8.09 % nitrogen, 41.61 % carbon, and 18.50 % sulfur, Elementar Analysensysteme GmbH, Hanau, Germany). The sample capsule was combusted at 900 °C with an excess of oxygen. A portion of the combustion gas was scrubbed of CO_2_, H_2_O, and SO_2_ and passed through a hot copper column at 500 °C to convert the NO_x_ forms to N_2_. All gases were removed by the appropriate traps leaving the analytically important CO_2_ and N_2_ which were subsequently detected with a thermal conductivity detector. The resulting N_2_ and CO_2_ gases were then measured by thermal conductivity in a high-purity helium carrier (Quality 5.0).

### Quantitative PCR

Quantitative RT-PCR analysis of a selected set of genes was performed. Locus IDs of these genes, some of which belong to multi-gene families, were selected based on differentially expressed proteins under drought in a proteome analysis conducted on Vandana and 481-B (Raorane et al. 2015). The proteome analysis suggested candidate genes; those that appeared especially relevant to the metabolite analysis were assessed at the transcript level in the three tissues of the three genotypes under well-watered and drought conditions. The primers were designed using Primer 3 (Supplementary Table 3). All the three tissues harvested for the metabolite measurements were rapidly pulverized into powder in liquid nitrogen and immediately transferred to TRIzol Reagent (Invitrogen, Life Technologies). The total RNA was then extracted according to the manufacturer’s recommendations. The cDNA was synthesized immediately after the RNA was extracted (ImProm-II™ Reverse Transcription System, Promega, USA). The quantitative PCR was performed with a Applied Biosystems 7500 Fast system (Applied Biosystems, USA) using the SYBR^®^ Select Real-time PCR Master Mix (Applied Biosystems, USA). The *C*_T_ values were determined with three technical replicates and normalized against cyclophilin expression (Δ*C*_T_) and using Way Rarem well-watered tissue as a reference sample (ΔΔ*C*_T_). The arithmetic averages were then calculated, and 2^−ΔΔ*C*T^ values (fold change) were used for the final visualization of the data.

### Statistical analysis

These analyses were performed based on various R packages (R Core Team 2013): For the Welch’s *t* test, the function was used; for PCA, the function prcomp was used; and for ANOVA and Tukey’s test, the corresponding functions described in the agricolae package were used (deMendiburu 2014).

An unpaired two-tailed Welch’s *t* test was applied to compare the means for unequal variances. *P* values of <0.05 were considered significant. A one-way ANOVA and Tukey’s honest significant differences (HSD) post hoc test were performed in all statistical analyses. Since tissues showed major significant differences among each other, analyses were separated by tissue to distinguish the drought-related variation and the genotypic-related variation. Differences with a *p* value of <0.05 were considered significant. Both the assumption of normality and the assumption of variance equality were tested with Shapiro–Wilks and Levene’s tests, respectively. For the principal component analysis, readings were separated in different data frames by tissue (*n* = 18) for comparison of metabolic profiles. Each data frame was analyzed separately. Two-dimensional score plots of the first two principal components were used to visualize the relative contribution of each individual metabolite to the clustering of the data between the 23 metabolites analyzed.

## Results

### Changes in the roots under drought

**Fig. 1:**
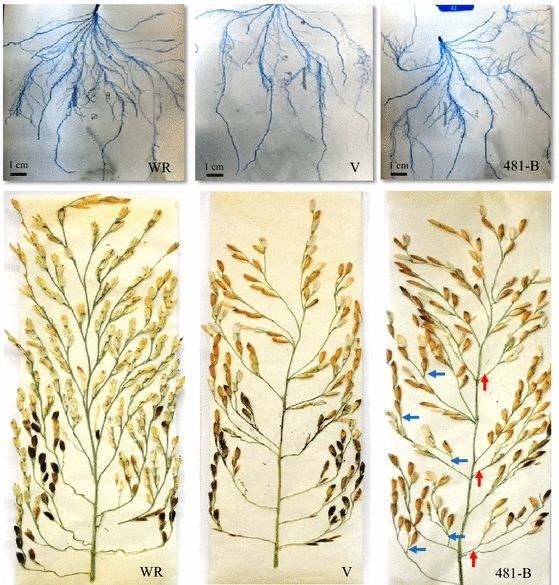
Root and panicle morphology of V, WR, and 481-B under drought. **a** Roots and **b** panicles of 481-B showed increased branching than V and WR. *Red arrows* (primary branches) and *blue arrows* (secondary branches) are used to indicate increased panicle branches in the NIL 481-B

Under PEG-simulated water-deficit conditions in vitro, increased lateral root growth and branching was observed in 481-B in comparison with Vandana and Way Rarem (Fig. 1a). Such drought-induced differences in root morphology were also notable in multiple field trials (Henry et al. 2014).

**Fig. 2:**
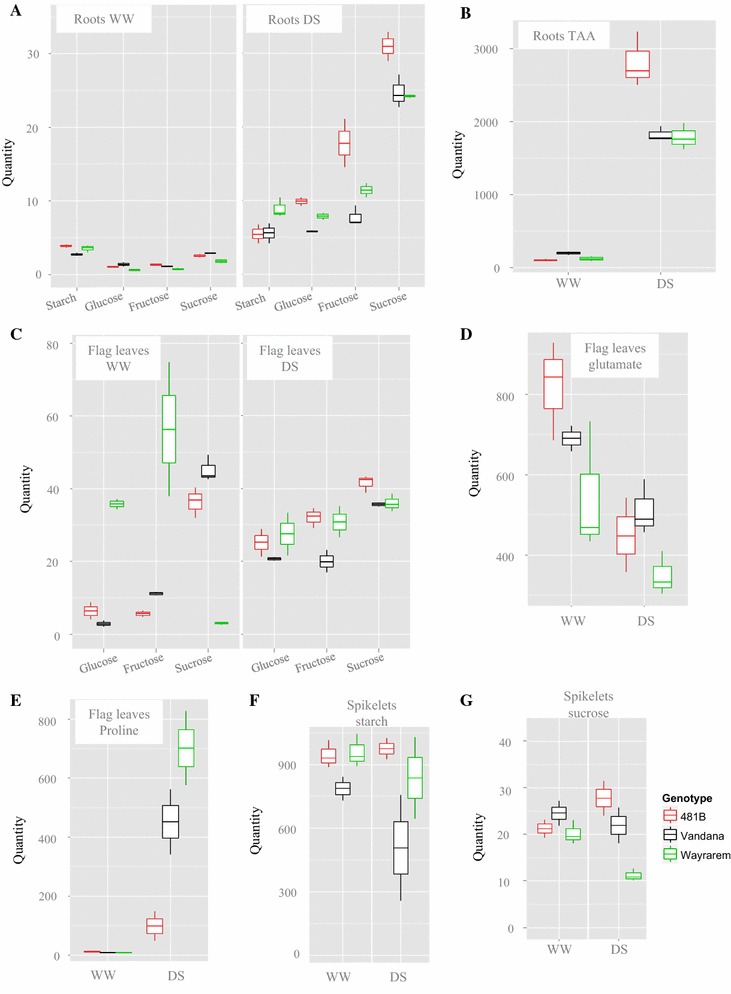
Boxplots of contents of specific metabolites in roots, flag leaves, and spikelets in well-watered and drought-stress conditions. **a** Boxplot of starch, glucose, fructose and sucrose in roots; **b** boxplot of total free amino acids in roots; **c** boxplot of glucose, fructose and sucrose in flag leaves; **d** boxplot of glutamate content in flag leaves; **e** boxplot of proline content in flag leaves; **f** boxplot of starch content in spikelets; **g** boxplot of sucrose content in spikelets. Components are represented in the horizontal axis and quantities in the vertical axis. Each *vertical white line* represents a metabolite or treatment as indicated, and the three boxes closest to it are the three genotypes: Vandana on the *white line* is coded in *black*, 481-B on the left of the *line in red*, and Way Rarem on the right of the *line in green* (as in the bottom right-hand side legend). In each *box*, the *horizontal line* represents the median of the data, the upper quartile represents the 75th percentiles, and the lower quartile represents the 25th percentile. The *whiskers* represented the 95 and 5 % of the data and the *circles*, when present, signified the outliers. Starch, glucose, fructose, and sucrose were measured in µmol/g fresh weight, while glutamate, proline, and total free amino acids were measured in pmol/mg

Roots of 481-B had greater amounts of sucrose, fructose, and glucose under drought conditions when compared to both Vandana and Way Rarem, but the starch content of 481-B was not significantly different than Vandana (Fig. 2a; Supplementary Figure 2). Way Rarem had the highest starch content increase in drought compared with well-watered conditions; in contrast, 481-B had the least starch increase under drought (Fig. 2a). Similarly, total free amino acid content was comparatively more in the roots of 481-B than in either parent under drought (Fig. 2b). However, the total free amino acid content increased in the roots of all three genotypes under drought (Fig. 2b). Individually, of the 16 amino acids analyzed, 13 were present in substantially larger amounts under drought in the roots of 481-B, while one, glutamate, was only marginally more abundant in 481-B roots (Supplementary Figure 2). Only two amino acids, isoleucine and GABA, were marginally more abundant in Way Rarem than in 481-B. However, even these two were present in a larger quantity in 481-B than in Vandana. The content of asparagine, aspartate, histidine, serine, glutamine, threonine, alanine, and proline was particularly increased under drought in the roots of 481-B (Supplementary Figure 2).

**Fig. 3:**
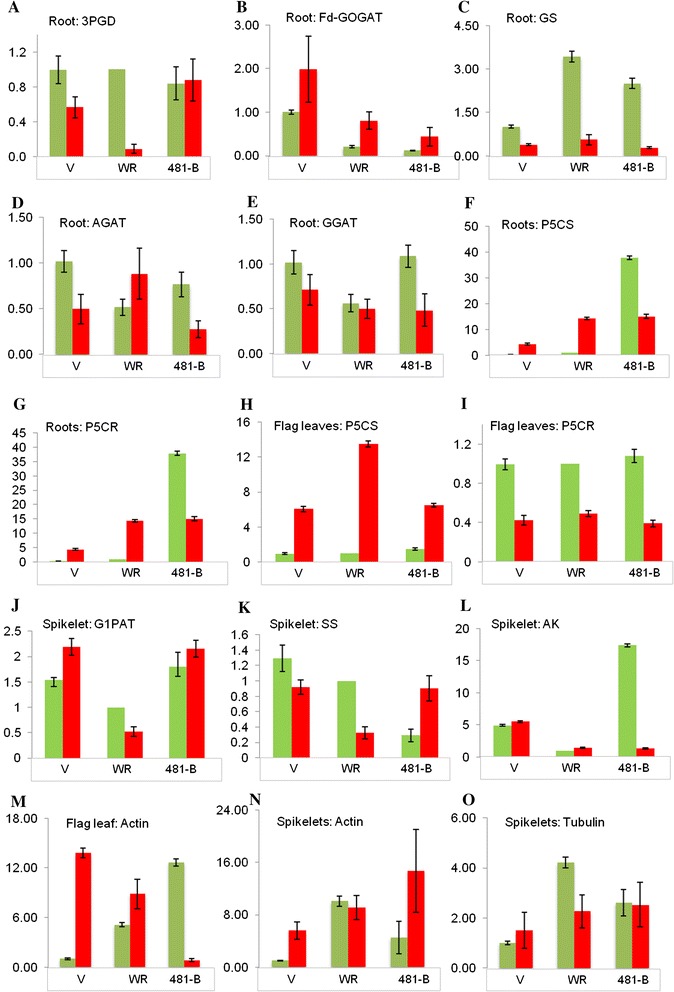
QRT-PCR results for selected amino acid synthesis genes. Genes involved in the metabolic pathways of serine, glutamate, glutamine, glycine, alanine, and proline metabolism were assessed for transcript abundance in the roots of Vandana (V), Way Rarem (WR), and the NIL 481-B. The *y* axis represents fold change in expression. *Bars in green* show transcript under well-watered conditions, and those in *red* indicate abundance under drought. The genes assessed were **a** 3-phosphoglycerate dehydrogenase (3PGD), **b** glutamine oxoglutarate aminotransferase (GOGAT), **c** glutamine synthetase (GS), **d** alanine glyoxalate aminotransferase (AGAT), **e** glutamate–glyoxylate aminotransferase (GGAT), **f**, **h** pyrroline 5-carboxylate synthetase (P5CS), **g**, **i** pyrroline-5-carboxylate reductase (P5CR), **j** glucose-1-phosphate adenylyltransferase (G1PAT), **k** sucrose synthase (SS), **l** adenylate kinase (AK), **m**, **n** actin and **o** tubulin. The average measurement and standard error is shown for each sample (*n* = 3)

Under drought, the dramatic increase of some amino acids in the roots generally but more specifically in 481-B (Supplementary Figure 2) was also reflected in the transcript level of some of the enzymes related to the metabolism of four of these amino acids. Transcript levels were assessed in all three genotypes under well-watered and drought conditions. In support of the higher content of serine in the roots of 481-B under drought, the transcript content of 3-phosphoglycerate dehydrogenase (3-PGD, EC 1.1.1.95), a key enzyme for serine biosynthesis, remained almost unchanged. 3-Phosphoglycerate dehydrogenase was significantly down-regulated in the roots of Vandana and Way Rarem (Fig. 3a). For glutamate content, the transcript of glutamate synthase (GOGAT, EC 1.4.1.13) in roots under drought increased fourfold in 481-B compared with twofold and threefold in Vandana and Way Rarem, respectively (Fig. 3b). Conversely, for glutamine synthetase (GS, EC 6.3.1.2), an enzyme that uses glutamate as a substrate, transcript levels were most reduced in 481-B (Fig. 3c), thus supporting metabolism toward a larger amount of glutamate. For alanine content, the transcript of alanine glyoxylate aminotransferase (AGAT, EC 2.6.1.44), which uses alanine to produce glycine and pyruvate, was down-regulated under drought in the roots of 481-B and Vandana but up-regulated in Way Rarem (Fig. 3d). Corroboratively, alanine content was lowest in Way Rarem and highest in 481-B. The transcript of glutamate–glyoxylate aminotransferase (GGAT, EC 2.6.1.4), which uses glutamate to produce glycine and 2-oxoglutarate, was also most down-regulated in the roots of 481-B, perhaps contributing to preserving the level of transportable glutamate (Fig. 3e). Unexpectedly for the comparatively larger content of proline in the roots of 481-B under drought (Supplementary Figure 2), the proline synthesis genes pyrroline 5-carboxylate synthetase (P5CS, EC 2.7.2.11) and pyrroline-5-carboxylate reductase (P5CR, EC 1.5.1.2) were both down-regulated in the roots of 481-B, while these were up-regulated in the roots of Vandana and Way Rarem (Fig. 3f, g). The importance and probable reasons of this paradox are discussed later.

### Changes in the flag leaves under drought

**Fig. 4:**
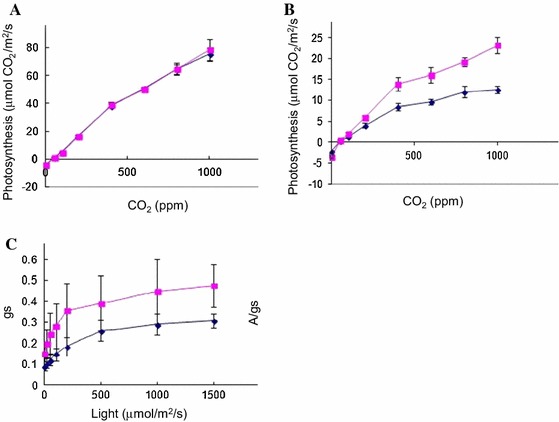
Comparison between Vandana and 481-B leaf characteristics. **a** Photosynthesis rate of 481-B and Vandana showed no difference under well-watered conditions, but under stress **b** 481-B had a lower photosynthesis rate than Vandana. The average measurement and standard error is shown for each of the samples (*n* = 3). **c** Stomatal conductance was lower for 481-B under stress. The average measurement and standard error is shown for each genotype (*n* = 3)

A more stable performance of 481-B across environments might be due to limited variation in the content of sugars (glucose, fructose, and sucrose) and starch in its flag leaves compared with higher variation in Vandana and Way Rarem (Fig. 2c; Supplementary Figure 3). Notably, the flag leaves of 481-B under drought had maximum sucrose content, and the combined amount of the three sugars (sucrose, glucose, and fructose) was greater in the flag leaf of 481-B (Fig. 2c). More sugars and less starch indicate decreased photosynthetic rate. Indeed, a similar photosynthetic rate between Vandana and 481-B under well-watered conditions (Fig. 4a) changed to a comparatively lower photosynthetic rate for 481-B under drought (Fig. 4b). Stomatal conductance under stress was more impeded in 481-B than in Vandana (Fig. 4c), which led to higher intrinsic water use efficiency (WUE) for 481-B compared with Vandana (Henry et al. 2014). The higher levels of sucrose, glucose, and fructose and lower levels of starch in 481-B may indicate a greater tendency to accumulate sugars under drought (Ambavaran et al. 2014) rather than the potential to accumulate newly assimilated photosynthate, since 481-B did not show higher photosynthesis rates than Vandana (Fig. 4b). Likewise, the lower stomatal conductance of 481-B points to the drought-response effect on WUE rather than assimilation ability, since it is mesophyll conductance rather than stomatal conductance that is more correlated with photosynthate assimilation and grain yield under drought in rice (Lauteri et al. 2014).

With regard to the amino acids, the flag leaves of all three genotypes showed high glutamate content under well-watered conditions, but it was the highest in 481-B (Fig. 2d). Under stress, 481-B apparently retained only about 50 % of the glutamate, while Vandana and Way Rarem retained about 73 and 65 % glutamate, respectively, in their flag leaves compared with that under well-watered conditions. Except for this reduction in glutamate in the flag leaves, the general pattern under stress was that of increased amino acids in the flag leaves and roots. However, the roots of 481-B under stress contained 14 and its flag leaves contained six amino acids (serine, threonine, alanine, lysine, valine, and leucine) at a greater level compared with the respective tissues of Vandana and Way Rarem. Other amino acids were present in lesser amounts in the flag leaves of 481-B than in Vandana and/or Way Rarem (Supplementary Figure 3). Unlike the roots, flag leaves of 481-B under stress contained significantly less proline than those of Vandana and Way Rarem (Fig. 2e; Supplementary Figure 3). Accordingly, the two proline synthesis genes, P5CS and P5CR, were also differentially regulated. P5CS was least up-regulated in 481-B during stress compared with Vandana and Way Rarem, suggesting lesser amounts of Δ1-pyrroline-5-carboxylate (P5C) which is one of the intermediates for proline synthesis. Furthermore, the enzyme (P5CR) involved in reducing P5C to proline was seen to be most down-regulated in the flag leaves of 481-B (Fig. 3h, i). This supported the metabolite data which indicated less proline in 481-B flag leaves during stress.

### Changes in the spikelets under drought

Under drought, the starch and sucrose contents in the spikelets of 481-B were more than in Vandana and Way Rarem (Fig. 2f, g; Supplementary Figure 4). Two starch biosynthesis enzymes important under drought, i.e., glucose-1-phosphate adenylyltransferase (EC 2.7.7.27) and sucrose synthase (EC 2.4.1.13) (Ahmadi and Baker 2001; Smith et al. 1997), were both more up-regulated in the spikelets of 481-B than in Vandana and Way Rarem (Fig. 3j, k). Similarly, in support of larger amounts of starch, which suggests reduced adenylate kinase (EC 2.7.4.3; Oliver et al. 2008), adenylate kinase was down-regulated in the spikelets of 481-B (Fig. 3l).

Similar to flag leaves and roots, there was a trend of increased total amino acid content under stress in the spikelets. Unlike roots, but similar to flag leaves, 481-B did not contain the highest amounts of most amino acids, but the two transportable amino acids, aspartate and glutamate, were present in the highest amounts in the spikelets of 481-B. Asparagine content was also high in 481-B, but lower in Vandana spikelets (Supplementary Figure 4). A trend for better N remobilization from the flag leaves to the spikelets of 481-B was suggested by the relatively larger amounts of transportable amino acids (asparagine, glutamine, aspartate, glutamate) in the flag leaves (Supplementary Figure 3) than the spikelets under well-watered conditions (Supplementary Figure 4). Under stress, however, the spikelets contained larger amounts of transportable amino acids. The trend of remobilization was also reflected in %N, C/N, and total free amino acid content when flag leaves and spikelets of the three genotypes were compared under well-watered and stress conditions (Supplementary Figures. 3, 4).

### Effect of *qDTY*_*12.1*_ on the performance of 481-B

The data were subjected to ANOVA to understand the significances in variation in primary metabolite content. Variation was analyzed by tissue to assess the effect of drought treatment (E) on the three genotypes (G) and their interaction (G × E). Significant differences were observed for G, E and G × E in most metabolites in all tissues studied (Supplementary Table 1). However, some differences were observed between tissues. For example, variation in the content of almost all metabolites in the flag leaves and roots was more influenced by G than by E. The G × E effect was least for most metabolites in the spikelets, i.e., spikelets of different genotypes under drought were more similar to each other than to the roots or flag leaves. In contrast, roots of the different genotypes under drought were least similar in metabolite content. The metabolite least affected by drought in the three tissues was glycine. Except for fructose in the flag leaf and sucrose in the spikelets, the content of glucose, fructose, and sucrose was prone to significant variation due to G, E, or G × E in all three tissues.

The Tukey’s post hoc test following the ANOVA showed that 481-B was significantly different from either parent for the content of specific amino acids in specific tissues and particularly for free sugars, and C in the roots. Under drought, both Way Rarem and 481-B had similar contents of valine and leucine in the flag leaves; histidine and starch in the spikelets; and aspartate, isoleucine, and phenylalanine in the roots. On the other hand, 481-B was significantly different from either parent for proline and lysine contents in the flag leaves; glutamate, glucose, and fructose contents in the spikelets and for up to 13 metabolites in the roots (Supplementary Table 2).

To better highlight such relationships, a Welch’s *t* test comparing Vandana and 481-B was performed. Variation in each metabolite in the three different tissues was assessed as the ratio of content under drought to well-watered conditions in order to reflect the genotypic response to drought and not only the genotypic differences (Tables 1, 2, 3). Overall, both genotypes showed different drought response for most amino acids in flag leaves and roots (Tables 1, 2). In the flag leaves, most differences involved various metabolites; however, the total free amino acid content did not change between Vandana and 481-B. Furthermore, the levels of carbohydrates, C, N, and their ratio remained unchanged (Table 1). On the other hand, in roots, the only metabolites with similar changes in both genotypes under drought were asparagine, glycine, GABA, starch, N, C, and C/N (Table 2). The composition of the tested set of metabolites in the spikelets of 481-B was similar to Vandana except for glutamate, isoleucine, phenylalanine, and glucose (Table 3).

**Table 1:** Results of Welch’s *t* test comparing the means of Vandana and 481-B for the 20 metabolites, total amino acids, carbohydrates, C, N, and C/N analyzed in flag leaf tissue

Component	*n*	Mean_V_	SD_V_	Mean_481B_	SD_481B_	*t*	*df*	*p* value	CI_.95_		Tukey HSD
His	6	14.02	7.71	12.26	1.60	−0.39	2.17	0.733	−19.92	16.39	
Asn	6	82.38	63.69	21.02	5.15	−1.66	2.03	0.237	−218.16	95.43	
Ser	6	1.35	0.11	3.28	0.20	14.83	3.18	0.000	1.52	2.32	***
Gln	6	7.75	3.77	2.69	0.95	−2.26	2.26	0.138	−13.74	3.62	
Gly	6	2.86	0.78	4.00	0.54	2.10	3.55	0.113	−0.45	2.73	
Asp	6	1.34	0.67	1.27	0.19	−0.19	2.31	0.863	−1.59	1.44	
Glu	6	0.74	0.08	0.54	0.03	−3.95	2.63	0.037	−0.37	−0.02	
Thr	6	2.02	0.49	3.87	0.03	6.51	2.02	0.022	0.63	3.05	**
Ala	6	0.84	0.19	2.37	0.39	6.16	2.96	0.009	0.73	2.33	**
GABA	6	2.87	0.98	4.22	1.78	1.15	3.10	0.331	−2.31	5.01	
Pro	6	54.65	12.03	8.79	4.63	−6.16	2.58	0.013	−71.89	−19.85	***
Lys	6	1.01	0.37	3.49	0.77	5.04	2.89	0.016	0.88	4.08	***
Val	6	2.19	1.10	5.18	0.24	4.60	2.18	0.037	0.41	5.57	***
Ile	6	9.13	5.13	4.07	0.99	−1.68	2.15	0.227	−17.20	7.08	
Leu	6	2.21	0.89	3.55	0.98	1.77	3.96	0.152	−0.77	3.47	***
Phe	6	1.46	0.41	4.76	1.02	5.20	2.63	0.019	1.11	5.49	*
Total aa	6	1.88	0.51	1.41	0.14	−1.52	2.32	0.250	−1.62	0.69	
Starch	6	2.54	0.37	1.05	0.11	−6.69	2.34	0.014	−2.32	−0.65	
Glucose	6	7.67	2.19	4.50	2.35	−1.71	3.98	0.163	−8.33	1.99	
Fructose	6	1.78	0.20	5.79	0.40	15.53	2.94	0.001	3.18	4.84	
Sucrose	6	0.79	0.07	1.15	0.17	3.34	2.62	0.054	−0.01	0.73	
N	6	0.89	0.17	0.89	0.15	0	3.96	1	−0.36	0.36	
C	6	1.19	0.02	1.25	0.06	1.63	2.36	0.226	−0.07	0.19	
C/N	6	1.38	0.32	1.41	0.20	0.17	3.37	0.875	−0.61	0.68	

Columns represent (from left to right): components; total number of samples analyzed (*n*); mean and standard deviation (SD) for Vandana and 481B, respectively (columns 3–6); Welch’s *t* value (*t*); degrees of freedom (*df*); *p* value; and the confidence interval at 95 % probability (CI_.95_). The values used for the calculation are the ratio of each component’s content in drought versus well-watered conditions, representing the genotypic response under drought. The last column represents the significance level obtained from the Tukey HSD test that compared Vandana, 481B, and Way Rarem (significance values are *, **, and *** representing 0.05, 0.01, and <0.001, respectively). Gray-shaded cells are components significant by Welch’s *t* test at *p* < 0.05. The comparison between the Welch’s *t* test and the Tukey HSD highlighted differences in the significance of some components

**Table 2:** Results of Welch’s *t* test comparing the means of Vandana and 481-B for the 20 metabolites, total amino acids, carbohydrates, C, N, and C/N analyzed in root tissue

Component	*n*	Mean_V_	SD_V_	Mean_481B_	sd_481B_	*t*	*df*	*p* value	CI_.95_		Tukey HSD
His	6	11.81	4.61	58.97	5.75	11.08	3.82	0.000	35.12	59.21	***
Asn	6	59.98	2.67	184.96	84.36	2.56	2.00	0.124	−84.28	334.23	
Ser	6	5.56	1.26	24.52	8.21	3.95	2.09	0.054	−0.81	38.74	**
Gln	6	4.82	2.21	20.83	2.90	7.61	3.74	0.002	10.00	22.01	***
Gly	6	1.20	0.45	1.62	0.57	1.01	3.79	0.374	−0.76	1.60	
Asp	6	6.54	0.28	20.87	3.71	6.68	2.02	0.021	5.19	23.46	*
Glu	6	4.28	1.10	8.68	0.62	6.04	3.14	0.008	2.14	6.66	***
Thr	6	13.27	3.26	34.47	16.08	2.24	2.16	0.145	−16.75	59.14	**
Ala	6	4.98	0.57	20.02	7.06	3.68	2.03	0.065	−2.35	32.42	**
GABA	6	2.52	0.39	6.60	2.18	3.19	2.13	0.079	−1.12	9.27	
Pro	6	46.65	16.35	143.67	16.41	7.26	4.00	0.002	59.89	134.14	***
Lys	6	14.36	2.60	52.84	13.61	4.81	2.15	0.035	6.22	70.75	***
Val	6	5.03	1.13	22.50	3.46	8.32	2.43	0.008	9.80	25.14	**
Ile	6	1.82	0.17	11.44	2.41	6.91	2.02	0.020	3.68	15.57	**
Leu	6	1.22	0.15	4.79	1.30	4.70	2.05	0.040	0.38	6.74	***
Phe	6	2.68	0.21	15.18	5.26	4.11	2.01	0.054	−0.54	25.54	***
Total aa	6	9.27	0.66	27.68	6.38	4.97	2.04	0.037	2.79	34.04	***
Starch	6	0.50	0.11	0.74	0.20	1.83	3.07	0.162	−0.17	0.64	
Glucose	6	4.44	0.75	9.95	1.33	6.24	3.14	0.007	2.77	8.24	***
Fructose	6	7.06	1.21	13.70	2.20	4.58	3.10	0.018	2.11	11.18	***
Sucrose	6	8.52	0.76	12.42	0.86	5.88	3.95	0.004	2.05	5.75	***
N	6	1.03	0.23	1.43	0.29	1.90	3.81	0.134	−0.20	1.00	
C	6	0.84	0.02	0.92	0.05	2.64	2.73	0.086	−0.02	0.17	***
C/N	6	0.84	0.19	0.66	0.10	−1.53	2.95	0.225	−0.58	0.21	

Columns represent (from left to right): components; total number of samples analyzed (*n*); mean and standard deviation (SD) for Vandana and 481B, respectively (columns 3–6); Welch’s *t* value (*t*); degrees of freedom (*df*); *p* value; and the confidence interval at 95 % probability (CI_.95_). The values used for the calculation are the ratio of each component’s content in drought versus well-watered conditions, representing the genotypic response under drought. The last column represents the significance level obtained from the Tukey HSD test that compared Vandana, 481B, and Way Rarem (significance values are *, **, and *** representing 0.05, 0.01, and <0.001, respectively). Gray-shaded cells are components significant by Welch’s *t* test at *p* < 0.05. The comparison between the Welch’s *t* test and the Tukey HSD highlighted differences in the significance of some components

**Table 3:** Results of Welch’s *t* test comparing the means of Vandana and 481-B for the 20 metabolites, total amino acids, carbohydrates, C, N, and C/N analyzed in spikelet tissue

Component	*n*	Mean_V_	SD_V_	Mean_481B_	SD_481B_	*t*	*df*	*p* value	CI._95_		Tukey HSD
His	6	2.71	1.05	1.88	0.48	−1.25	2.79	0.304	−3.04	1.37	***
Asn	6	11.93	4.91	19.07	2.33	2.27	2.86	0.112	−3.14	17.41	
Ser	6	1.65	0.35	1.98	0.96	0.55	2.52	0.630	−1.78	2.43	
Gln	6	2.10	0.17	3.61	1.04	2.49	2.11	0.125	−0.98	4.01	
Gly	6	5.09	1.99	6.31	1.07	0.94	3.06	0.416	−2.88	5.33	
Asp	6	1.07	0.26	1.43	0.31	1.57	3.90	0.194	−0.29	1.01	
Glu	6	0.98	0.22	1.71	0.30	3.43	3.66	0.031	0.12	1.34	*
Thr	6	1.21	0.28	1.99	0.44	2.60	3.42	0.070	−0.11	1.67	
Ala	6	0.87	0.28	1.05	0.23	0.86	3.84	0.442	−0.41	0.77	
GABA	6	4.40	2.62	2.72	0.22	−1.10	2.03	0.383	−8.11	4.76	
Pro	6	36.62	14.05	22.70	1.54	−1.71	2.05	0.227	−48.25	20.42	
Lys	6	3.12	1.17	2.52	0.96	−0.69	3.85	0.532	−3.07	1.87	
Val	6	0.68	0.05	1.13	0.35	2.18	2.07	0.156	−0.41	1.30	
Ile	6	0.61	0.03	1.26	0.19	5.69	2.07	0.027	0.17	1.12	
Leu	6	0.88	0.15	0.95	0.30	0.35	2.92	0.750	−0.55	0.68	
Phe	6	0.83	0.19	1.62	0.28	4.00	3.50	0.021	0.21	1.37	
Total aa	6	2.36	0.60	2.64	0.55	0.60	3.97	0.581	−1.02	1.58	
Starch	6	1.87	1.03	0.97	0.12	−1.49	2.05	0.271	−3.42	1.63	*
Glucose	6	3.14	0.76	1.40	0.26	−3.74	2.44	0.047	−3.43	−0.05	***
Fructose	6	3.96	1.41	1.65	0.31	−2.77	2.19	0.099	−5.62	1.00	***
Sucrose	6	0.89	0.14	1.32	0.27	2.46	2.99	0.091	−0.13	0.97	
N	6	0.94	0.08	1.19	0.16	2.40	2.86	0.100	−0.09	0.58	
C	6	1.06	0.01	1.09	0.06	0.83	2.04	0.495	−0.11	0.16	
C/N	6	1.13	0.09	0.92	0.08	−3.04	3.96	0.039	−0.40	−0.02	

Columns represent (from left to right): components; total number of samples analyzed (*n*); mean and standard deviation (SD) for Vandana and 481B, respectively (columns 3–6); Welch’s *t* value (*t*); degrees of freedom (*df*); *p* value; and the confidence interval at 95 % probability (CI_.95_). The values used for the calculation are the ratio of each component’s content in drought versus well-watered conditions, representing the genotypic response under drought. The last column represents the significance level obtained from the Tukey HSD test that compared Vandana, 481B, and Way Rarem (significance values are *, **, and *** representing 0.05, 0.01, and <0.001, respectively). Gray-shaded cells are components significant by Welch’s *t* test at *p* < 0.05. The comparison between the Welch’s *t* test and the Tukey HSD highlighted differences in the significance of some components

Comparing the results of both the Welch’s *t* test and the Tukey’s HSD showed similar results in roots (Table 2, last column); however, flag leaves and spikelets showed differences between the two statistical approaches. However, both tests revealed that differences under drought were more pronounced in the roots and flag leaves than in the spikelets (Tables 1, 2, 3).

**Fig. 5:**
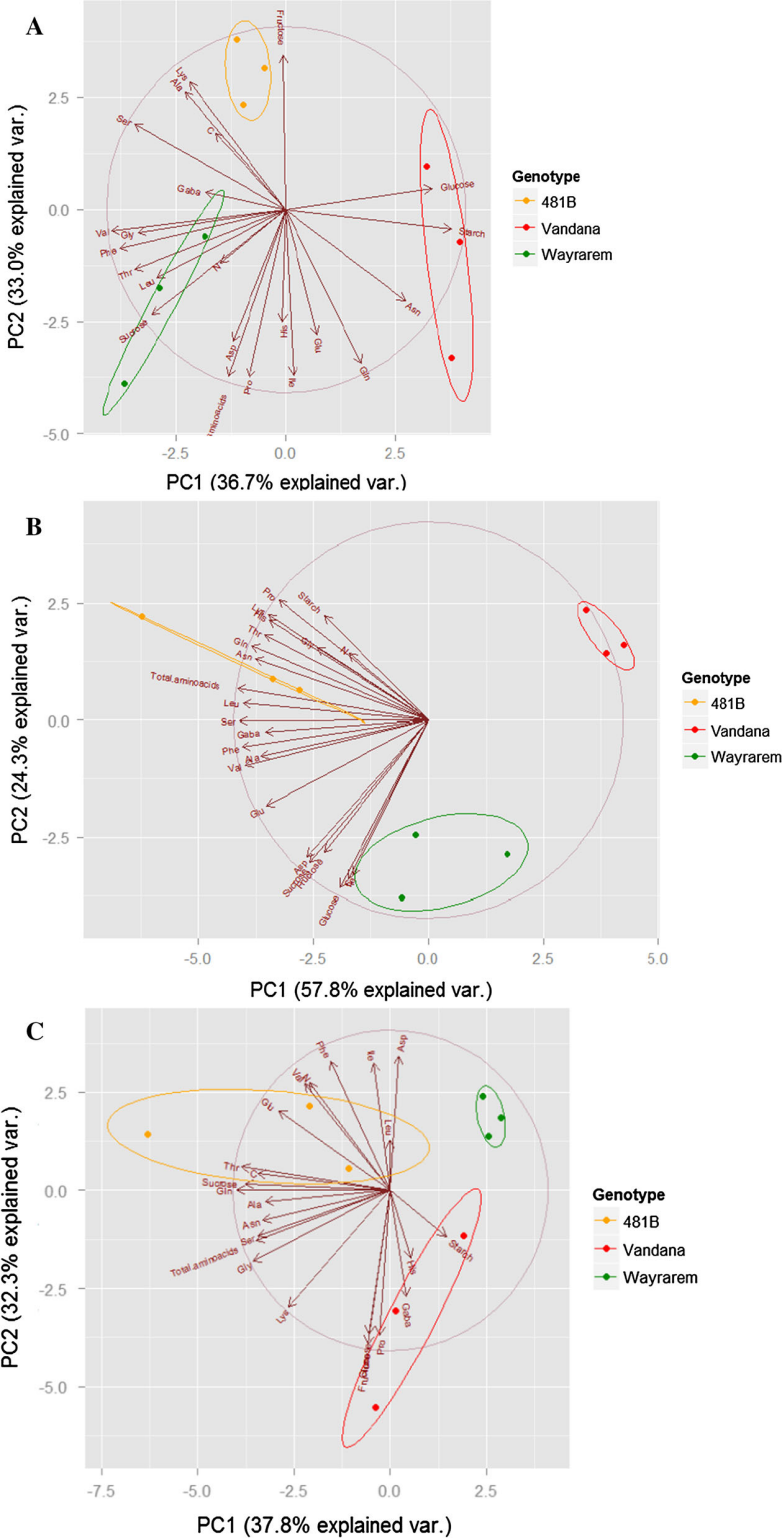
Principal component analysis (PCA) of different metabolites in **a** flag leaves, **b** roots, and **c** spikelets in their response to drought stress. A common set of 23 metabolites is represented for each genotype. Each data point represents the ratio of the metabolite content in drought conditions divided by the metabolite content in well-watered conditions in each genotype. Vandana is represented in orange, 481-B is in *red*, and Way Rarem is in *green*. The *arrows* represent the vectors for each metabolite, the direction varying toward the highest squared multiple correlation with the principal component

Contributions of *qDTY*_*12.1*_ per se were better highlighted by PCA. In an analysis of the ratio of metabolite content in well-watered and drought conditions, the separation pattern of the three genotypes was very different for each of the three tissues. This indicated a differential response to drought by each genotype in each tissue. However, for the flag leaves, NIL 481-B clustered close to Way Rarem in relation to PC1 (Fig. 5a). The case was similar for the positive components of PC2 (fructose, lysine, and alanine). NIL 481-B was between Vandana and Way Rarem for the negative components of PC2 (total amino acids, proline, histidine, isoleucine, glutamine, glutamate, and aspartate). In roots, the negative component of PC1 and PC2 was likely affected by Way Rarem and 481-B, while the positive components of PC2 were affected by Vandana (Fig. 5b). In spikelets, 481-B showed more similarities with Way Rarem in both PCs (Fig. 5c), but especially in PC2 (starch, glucose, asparagine, total amino acids, aspartate, glutamate, glutamine, isoleucine, and histidine). *qDTY*_*12.1*_ apparently affected particular metabolic pathways more than particular tissues. Most of the PCs were composed of proline, histidine, isoleucine, total amino acids, starch, and glucose. Additionally, some amino acids grouped together in all tissues (alanine and serine, and phenylalanine and valine).

## Discussion

### Lateral roots and root metabolites

Increased lateral root proliferation is implicated in plant sustenance under drought (Zhu et al. 2005; Chapman et al. 2012) as roots help in the continued acquisition of water and nutrients. In this study, 481-B showed marked differences in root branching and root metabolite content when compared to the parental lines Vandana and Way Rarem. The difference in root morphology was also notable in PEG-simulated water-deficit conditions in vitro. Such a morphological change in lateral root profusion in 481-B under drought has been found to be stable across environments (Henry et al. 2014). This suggests that the rapid profusion of lateral roots under drought in 481-B but not in Vandana or Way Rarem is most likely not a manifestation of G × E but of G × G interactions.

During drought, N balance is of high importance to the plant (Kohli et al. 2012). In rice under drought, growth and sustenance are better in plants with N supply than in those without (Suralta 2010). Irrespective of the soil N status, rice roots show increased lateral roots under drought, but drought-tolerant cultivars exhibit even more lateral roots (Suralta 2010). Since N absorption is limited during drought, increased lateral roots in 481-B may result in increased capacity to extract N from the soil.

A high correlation between localized hexose concentration and fast-growing, highly branched roots is known (Freixes et al. 2002). High hexose concentration increases respiration rates in wheat roots (Bingham and Stevenson 1993). The finding of less starch but more sugars in the roots of 481-B compared with the two parents may be useful toward lateral root growth, for fulfilling the concomitant energy needs and/or synthesizing the structural components such as cellulose. High starch in Vandana roots may not be helpful, just as starch accumulation in roots under drought stress is correlated with impaired growth in a variety of plants (Galvez et al. 2011).

The content of asparagine, aspartate, histidine, serine, glutamine, threonine, alanine, and proline was particularly increased by drought and comparatively greater in the roots of 481-B than in both Vandana and Way Rarem. An overall increase in amino acids under drought is known in rice leaves but correlated with drought-sensitive cultivars (Degenkolbe et al. 2013). The functional importance of the variable content of easily transportable amino acids such as glutamate and aspartate is known.

Serine is implicated in plant responses to various environmental stresses (Ho and Saito 2001). The enhanced content of serine in Arabidopsis by overexpressing the 3-PGD gene leads to enhanced tolerance to different abiotic stresses (Waditee et al. 2007). The high serine content in 481-B may also be involved in the generation of other essential amino acids such as glycine, cysteine, and methionine (Ufaz and Galili 2008), which may be useful components of stress tolerance.

Alanine accumulation in roots is known in *Medicago truncatula* under flooding (Limami et al. 2008), in soybean and Arabidopsis under hypoxia and anoxia (Miyashita et al. 2007; Rocha et al. 2010), and in wheat shoots under water deficit (Drossopoulos et al. 1985). However, reduction in alanine occurs in roots of moderately drought-tolerant barley under drought (Sicher et al. 2012). Alanine is a substrate for alanine glyoxylate aminotransferase (AGAT), one of the products of which is pyruvate, the only readily available source of carbon skeleton during stress (Good et al. 2007). Less alanine and concomitantly increased content of AGAT may equate to the need for more pyruvate in Way Rarem, suggesting its drought susceptibility.

Glutamate synthase or glutamine oxoglutarate aminotransferase (GOGAT) increased fourfold in 481-B under drought compared with twofold and threefold in Vandana and Way Rarem, respectively (Fig. 3b). Glutamate is one of the transportable amino acids, as well as a precursor for the synthesis of other amino acids such as arginine and proline (Ramanjulu and Sudhakar 1997). A general down-regulation of glutamine synthetase (GS) and up-regulation of GOGAT in the roots of 481-B and the parents putatively reflected the need for a high amount of glutamate under stress (Fig. 3b, c). However, in 481-B roots, both glutamate and glutamine increased under stress (Supplementary Figure 2), which led to the desirable higher glutamine/glutamate ratio compared with Vandana and Way Rarem. A high glutamine/glutamate ratio in plants is indicative of a good N status and of a desirable balance between the C and N compounds (Swarbreck et al. 2011). The trend of maintaining a high glutamine/glutamate ratio under stress was also evident through down-regulation of GGAT to the greatest degree in the roots of 481-B (Fig. 3e). The glutamine and glutamate metabolism pathway is of crucial importance since the two molecules contribute to the biosynthesis of major N-containing compounds including amino acids, nucleotides, chlorophylls, polyamines, and alkaloids (Lea and Ireland 1999).

Along with glutamine, proline is an important N storage amino acid. Proline accumulation in the roots of drought-tolerant rice cultivars is known (Hien et al. 2003), but its function is not fully understood. Proline is a water-soluble amino acid, which exists as a zwitterion that accumulates under dehydration and has roles in cellular osmolarity, redox potential buffering, and energy transfer as per cellular demands depending on spatiotemporal control of its metabolism (Szabados and Savouré 2010; Verslues and Sharma 2010). Such properties depend not only on proline itself but also on its metabolic cycle. Glutamate is the source of proline synthesis through the enzymes P5CS and P5CR. Limited understanding exists of the role of proline for crop yield under drought. “The more the better” is the popular concept for proline under stress, but higher accumulation of proline has never shown any direct correlation with yield under stress. High amounts of proline in the roots of 481-B without the concordant increase in proline synthesis gene transcripts, and a simultaneous low amount of proline in its flag leaves led to the hypothesis that proline in 481-B was translocated from the leaves to the roots during stress. Colorimetric measurement during stress revealed much larger amounts of proline in the stem of 481-B than in that of Vandana and Way Rarem (Supplementary Figure 1), thus supporting the hypothesis. Proline supply from the shoot and its catabolism in the root is essential for continued growth at low water potential in *Arabidopsis* (Sharma et al. 2011). Maize root tips also accumulate high levels of proline during stress due to translocation and not due to de novo synthesis (Verslues and Sharp 1999). Such observations suggest that stress-induced tissue-specific differences in proline metabolism and accumulation may be more important than previously thought. The roots of 481-B thus seem better equipped to counter stress and perform their critical functions toward drought tolerance. Whether or not proline accumulation is a stress indicator or part of an adaptive response has long been debated. Earlier research correlates proline to stress, and thus, higher proline accumulation was taken to indicate a higher degree of stress (Stewart and Hanson 1980). However, higher proline content was recently associated with better drought and salt tolerance (Ben Hassine et al. 2008; Evers et al. 2010; Kant et al. 2006). Reverse genetics and other molecular approaches demonstrate that stress-induced proline accumulation is useful; however, these approaches also provide further insights into other possible functions of proline such as maintenance of homeostasis (Kishor and Sreenivasulu 2013). Decrease in soil water potential leads to higher levels of proline in unvacuolated cells and root tips for osmotic adjustment (Verslues and Sharp 1999; Bussis and Heineke 1998), whereby cellular structures and membranes are stabilized through hydrophilic interactions and hydrogen bonding to maintain turgor pressure and water content (Verslues and Sharma 2010). In the case of 481-B, drought-induced lateral root growth may gain from the combined capacity of proline to act as a redox buffer, as a source of energy, and as an osmoticum to maintain due cellular homeostasis and tissue functionality. A proteomic study comparing Vandana and 481-B under drought revealed differential content of enzymes related to serine, glutamate, alanine, and proline metabolism in the roots (Raorane et al. 2015), thus supporting the findings from this study on concomitant changes in the content of these four amino acids in the roots. These results suggest a relationship between the altered content of these amino acids and *qDTY*_*12.1*_.

### Variation in primary metabolites in the flag leaves

Under well-watered conditions, the flag leaves of all three genotypes showed high glutamate content, but it was the highest in 481-B (Fig. 2d). However, NIL 481-B had less glutamate than Vandana in its flag leaves under stress. In parallel, well-watered spikelets of 481-B contained less glutamate than Vandana, but under stress 481-B contained more glutamate than Vandana (Supplementary Figure 4). Transportable glutamate plays a key role in N remobilization, which is an important aspect of plant development and survival under stress. The rate of N remobilization increases when N absorption is limited due to water deficit (Masclaux-Daubresse et al. 2008). The large amount of glutamate in 481-B flag leaves could potentially be remobilized to its spikelets under drought. Remobilized amino acids may serve as precursors feeding into other anabolic pathways. They play a major anaplerotic role to feed the intermediates of the TCA cycle (Ashraf and Foolad 2007; Sweetlove et al. 2010), act as osmolytes (Verslues and Sharma 2010), and define the overall N status under drought.

Increased alanine in the flag leaves of 481-B, as in its roots, was against the observation that alanine decreases in the leaves of drought-tolerant rice genotypes under drought (Degenkolbe et al. 2013). In chickpea, alanine decreases in drought-sensitive genotypes under drought (Thavarajah and Ball 2006), while in soybean the drought-tolerant as well as the drought-sensitive genotypes exhibit decreased leaf alanine under water stress (Silvente et al. 2012). In the case of flag leaves of 481-B under drought, a potential reason for alanine accumulation might be the reduced amount of the alanine glyoxylate aminotransferase (AGAT; Fig. 3d). This in turn might be a consequence of a lack of need for the breakdown of alanine into pyruvate to supply the carbon skeletal for anabolic reactions.

The reduced content of most amino acids in flag leaves under drought was generally in keeping with the reported negative correlations between most amino acids, including asparagine, glutamate, glutamine, and glycine, and rice plant performance under drought (Degenkolbe et al. 2013). For example, asparagine content is negatively correlated with WUE under drought (Degenkolbe et al. 2013). WUE being better in 481-B than Vandana (Henry et al. 2014), it correspondingly contained less asparagine than Vandana under drought (Supplementary Figure 3). Degenkolbe et al. (2013) suggested that higher amounts of serine and threonine in the leaves under well-watered conditions are suggestive of drought-tolerant genotypes. However, flag leaves of the relatively more tolerant 481-B contained less serine than Vandana (Supplementary Figure 3). Such a discrepancy could be due to genotypic differences related to the parental subpopulations (Way Rarem is *indica* and Vandana is *aus*), differences in ecosystems (QTL *qDTY*_*12.1*_ is mostly effective in upland conditions), leaf tissue (we analyzed flag leaves, compared with other studies that use leaves formed during vegetative stage), and developmental stage (pre-booting stage compared to post-fertilization stage). These results suggest that identifying metabolites as markers for breeding stress-tolerant cultivars will depend on highly standardized protocols considering tissue type, developmental stage, type of stress, plant ancestry, and perhaps other agroecological parameters.

An increased glycine/serine ratio is indicative of enhanced photorespiration (Novitskaya et al. 2002), which is related to a higher degree of stress; the higher the ratio, the more susceptible the genotype. Among the three genotypes studied, Way Rarem is the most drought susceptible. Its flag leaves contained more glycine and relatively less serine under drought; hence, the glycine/serine ratio was higher in Way Rarem flag leaves compared with Vandana and 481-B (Supplementary Figure 3).

The combined amount of the three sugars (sucrose, glucose, and fructose) was greater in the flag leaves of 481-B than in those of Vandana or Way Rarem. Notably, the flag leaves of 481-B under drought had the greatest sucrose content, while the starch content was more than in Vandana but less than in Way Rarem. In leaves, soluble sugars accumulate under stress and function as metabolic resources, structural component substrates, and signaling molecules in processes associated with growth and development (Jang and Sheen 1997; Tran et al. 2007; Ho et al. 2001). Under drought, sucrose and glucose serve as substrates for osmolyte synthesis toward maintaining homeostasis by protecting membranes, enzymes, and other structures against damage and denaturation (Gupta and Kaur 2005). They are also used in respiration to meet cellular energy needs when photosynthesis is typically down-regulated to conserve energy and water under drought (Pinheiro and Chaves 2011). Fructose, however, can be converted to erythrose-4-phosphate, which is involved in the synthesis of secondary metabolites such as phenolic compounds (Hilal et al. 2004) that have an important role under drought (Weidner et al. 2009). Such usefulness of sugars and their relative abundance in 481-B reiterated their association with drought tolerance. However, although Vandana is more drought tolerant than Way Rarem, its flag leaves did not show more soluble sugars than in Way Rarem under drought. Overall, the change in trends of sugar content between well-watered and drought conditions suggests the glucose and fructose contents of 481-B under drought to be influenced by the drought response of Vandana, while the sucrose content was influenced by Way Rarem. Flag leaf starch content in Way Rarem and 481-B remained rather similar under well-watered and drought conditions, but it was considerably reduced in Vandana under drought.

Sugar and starch contents are influenced by the rate of photosynthesis, which is classically reduced under stress (Chaves 1991). A main determinant of reduced photosynthesis under drought is reduced stomatal opening, which consequently hampers CO_2_ entry for fixation by photosynthesis but also avoids loss of water through reduced stomatal conductance. Partial stomatal closure can restrict stomatal conductance while allowing limited CO_2_ entry for a relatively functional level of photosynthesis (Flexas and Medrano 2002). Such a compromise leads to improved intrinsic water use efficiency (WUE) as exemplified by 481-B. For stomatal closure, the status of actin is a governing factor (Lemichez et al. 2001), and the kind and amount of actin influences stomatal opening (Hwang et al. 1997; Kim et al. 1995; Lemichez et al. 2001). Actin gene expression of LOC_Os01g64630 was more down-regulated in the flag leaves of 481-B compared with Vandana and Way Rarem (Fig. 3m). Whether or not this is a critical change toward preventing complete stomatal closure and thus better WUE and photosynthetic efficiency requires further studies because there are 21 other actin genes whose expression could be affected.

### Grain yield and spikelet metabolites

Grain yield is based on N uptake before flowering and on the remobilization of N during seed maturation (Kichey et al. 2007). Amino acids play a crucial role in N usage by plants through the processes of uptake, assimilation, translocation, and remobilization (Masclaux-Daubresse et al. 2008). The content of easily transportable amino acid glutamate is a measure of N remobilization. Glutamate content was indeed greater in the flag leaves of 481-B under well-watered conditions, but its content was reduced under drought (Supplementary Figure 3). In spikelets, glutamate content was less in 481-B than in Vandana under well-watered conditions but higher than in Vandana under drought (Supplementary Figure 4). One reason for this could be the more efficient remobilization of glutamate from flag leaves to the spikelets under drought in 481-B. A similar remobilization may be expected from the roots where glutamate was more abundant in 481-B (Supplementary Figure 2). Ammonia produced by nitrate reduction, amino acid degradation, and photorespiration is used along with glutamate for the synthesis of glutamine by glutamine synthetase (GS). To enable glutamate accumulation and remobilization, GS should be correspondingly reduced. This was indeed observed in 481-B (Fig. 3c). Along with the high content of glutamate in the flag leaves and roots of 481-B and the possibility of its better remobilization to the spikelets under stress, other transportable amino acids were present in comparatively large amounts in the spikelets of 481-B. This study (Fig. 1b) and that of Henry et al. (2014) both indicated a greater number of total spikelets per panicle as well as the number of filled spikelets per panicle in 481-B under drought compared with Vandana and Way Rarem. This suggests better growth and development of the spikelets in 481-B.

Cytoskeleton proteins such as actins and tubulins play an important role in developing spikelets. Actins affect cell growth in the spikelets (Hussey et al. 2006), while tubulins also respond to flowering and spikelet development (Sheoran et al. 2014; Giani and Brevario 1996). An actin and tubulin were found to be more abundant in the spikelets of 481-B than in Vandana through a proteomic study (Raorane et al. 2015). Comparison of the transcript of the particular actin and tubulin in the spikelets of the three genotypes revealed that the actin was more upregulated under drought in 481-B and Vandana compared Way Rarem (Fig. 3n). Similarly, the tubulin transcript was drastically down-regulated in Way Rarem but not in Vandana and 481-B (Fig. 3o). As in the flag leaves, these results do not account for the expression of many other actin and tubulin genes; however, the hypothesis would be that actins and tubulins might be a part of the reason why spikelet development suffered in Way Rarem and that the combined effect of these cytoskeleton proteins required for growth and development may put 481-B at an advantage for spikelet development compared with Vandana.

### Effects of qDTY_12.1_

The three genotypes used in the study formed a potentially very informative panel because *qDTY*_*12.1*_ was identified from the susceptible parent Way Rarem (Bernier et al. 2007). The interesting question to be answered was: To what extent is the drought tolerance of 481-B influenced by the Way Rarem donor segment of *qDTY*_*12.1*_ or by the tolerant recipient genome of Vandana?

For the PCA, the hypothesis was that the contrasting parental genotypes Vandana and Way Rarem would exhibit variable response to drought at the metabolite level and would thus cluster in separate regions. We also expected that 481-B would lie close to Vandana for a particular tissue if *qDTY*_*12.1*_ did not affect metabolites, or it would cluster closer to Way Rarem if it did. PCA by tissue confirmed these hypotheses. ANOVA suggested that the variation in the content of almost all metabolites in the flag leaves and roots was more influenced by G than by E. The analysis also showed that 481-B was significantly different from both parental lines. Overall, Tukey’s post hoc test revealed that genotypes had different responses to drought for most amino acids in the different tissues. Such G-based variation implies that the response of 481-B was not dictated by the Vandana genome or the introgressed Way Rarem genes but perhaps by their interaction. Welch’s *t* test reinforced the idea that 481-B and Vandana have very different metabolic content, particularly in roots. In addition, in the PCA of the ratio of metabolite contents in well-watered to drought conditions, the three genotypes were separated and the separation pattern was very different for each of the three tissues. This again suggests that each genotype had a differential response to drought and that the response was different in each tissue. The differences in metabolite variation in 481-B to either parent in the different tissues, which was more pronounced in the roots, reiterated the epistatic interactions suggested by ANOVA and confirmed the previous finding of epistatic interactions of *qDTY*_*12.1*_ (Dixit et al. 2012).

The close clustering of 481-B to Way Rarem in relation to PC1 in flag leaves may imply that in 481-B, the positive (starch, glucose, and asparagine) and the negative components (valine, phenylalanine, serine, threonine glycine, leucine, and sucrose) of PC1 were most likely affected by the introgression of *qDTY*_*12.1*_. Similarly in PC2 (fructose, lysine, and alanine), Way Rarem and 481-B clustered together. In roots, the negative component of PC1 and PC2 was likely affected by the introgression of *qDTY*_*12.1*_, while the positive components of PC2 were affected by Vandana. Apparently *qDTY*_*12.1*_ affected particular metabolic pathways more than particular tissues. Most PCs were composed of proline, histidine, isoleucine, total amino acids, starch, and glucose.

Proximity of 481-B to Way Rarem for PC1 and PC2 in the spikelets suggested that *qDTY*_*12.1*_ had a particular effect on metabolites in the spikelets at reproductive stage. This suggestion from the PCA was somewhat contrary to the message from both the Welch’s *t* test and Tukey’s HSD that metabolites in the 481-B spikelet were not different from either parent. Thus, it may be that changes mediated by *qDTY*_*12.1*_ in roots and flag leaves indirectly affected, most likely through remobilization of metabolites, minimal changes in the spikelets.

## Summary and conclusions

Comparison of variations in the contents of a limited set of primary metabolites in three different tissues of three genotypes, a *qDTY*_*12.1*_ NIL and the two parental lines, indicated that under drought there were variations in a larger number of metabolites in roots than in the flag leaves and spikelets. Alterations of metabolites in 481-B roots were significantly different from those in the two parental lines. However, the content of most metabolites in 481-B spikelets was not significantly different from the parents, while that of 481-B flag leaves was more similar to Way Rarem than Vandana. These results imply that epistatic interaction between *qDTY*_*12.1*_ and the Vandana genome affects the roots in 481-B and that *qDTY*_*12.1*_*per se* significantly affects flag leaf metabolites. Therefore, changes in the roots and flag leaves largely formed the basis for the changes in the spikelets toward yield under stress, especially for amino acids.

Differences in amino acid remobilization in 481-B compared with Vandana and Way Rarem must underlie the effectiveness of *qDTY*_*12.1*_. For example, the high amounts of leaf glutamate and proline might putatively be transported to spikelets and roots, respectively. Additionally, efficient conversion of the drought-induced increase in spikelet glucose, fructose, and sucrose into starch underscored the increase in yield under stress of 481-B. Less starch and much more free sugars were detected in Vandana and Way Rarem. The effects of G, E, and G × E on the content of the three sugars varied significantly in the three tissues. However, the fructose and sucrose contents in 481-B spikelets were not significantly different from Way Rarem and Vandana, while the glucose content was significantly different from both. The increase of sugars in the spikelets was apparently related to Vandana, while its conversion to starch was apparently related to Way Rarem. Thus, *qDTY*_*12.1*_ had a role in positively affecting grain filling in 481-B.

The C/N ratio in each tissue was similar between the tolerant and susceptible genotypes. Although the content of metabolites varied, the QTL had no apparent effect on total C and N percentage in any tissue. This reiterated the PCA result that the QTL affected the metabolic pathways in the tissues rather than the tissue itself. The manifestation of such altered metabolic pathways might thus be the enhanced lateral root and panicle branching, water use efficiency, and grain filling. Nevertheless, the altered metabolic pathways were a consequence of G × E and G × G interactions rather than of the single dominant gene effect of the tolerant parent.

This summary of results led us to conclude that screening for drought tolerance was different from screening for yield under drought. Compared with 481-B, Vandana is a tolerant but lower yielding variety under drought, which apparently accumulated free sugars but perhaps lacked the capacity to remobilize and convert them into starch. Way Rarem apparently lacked enough free sugars but putatively harbored within *qDTY*_*12.1*_ the capacity to effectively convert them into starch. The introgression of *qDTY*_*12.1*_ thus makes 481-B better yielding under drought.

It was also evident that although yield under drought was the screen for *qDTY*_*12.1*_ and not the secondary traits related to roots, leaves, or panicles, it was actually the modifications of such secondary traits morpho-physiologically (lateral roots, leaf TE, and panicle branching) and metabolically (changes in root and leaf metabolites) that led to better yield under stress, although the spikelets per se remained metabolically similar to the parental genotypes. Such morpho-physiological and biochemical phenotypes by themselves might not be a guarantee for improved yield under stress, however, as exemplified by the lack of rice varietal development through secondary traits. Hence, it is important to primarily screen for better yield under drought.

Finally, for gainful insights into drought tolerance mechanisms toward yield under stress, multiple tissues and genotypes must be assessed at specific time points of development to avoid misleading conclusions for using particular genes/proteins/metabolites as markers for drought tolerance.

## Electronic supplementary material

Supplementary material 1 (PDF 570 kb)

## References

[CR1] Ahmadi A, Baker DA (2001). The effect of water stress on the activities of key regulatory enzymes of the sucrose to starch pathway in wheat. Plant Growth Regul.

[CR2] Ambavaran MMR, Basu S, Krishnan A, Ramegowda V, Batland U, Rahman L, Baisakh N, Pereira A (2014). Coordinated regulation of photosynthesis in rice increases yield and tolerance to environmental stress. Nat Commun.

[CR3] Ashraf M, Foolad MR (2007). Roles of glycine betaine and proline in improving plant abiotic stress resistance. Environ Exp Bot.

[CR82] Bates LS, Waldren RP, Teare ID (1973) Rapid determination of free proline for water-stress studies. Plant Soil 39:205–207

[CR4] Ben Hassine A, Ghanem ME, Bouzid S, Lutts S (2008). An inland and a coastal population of the Mediterranean xero-halophyte species *Atriplex halimus* L. differ in their ability to accumulate proline and glycine betaine in response to salinity and water stress. J Exp Bot.

[CR5] Bernier J, Kumar A, Venuprasad R, Spaner D, Atlin G (2007). A large-effect QTL for grain yield under reproductive stage drought stress in upland rice. Crop Sci.

[CR6] Bernier J, Kumar A, Venuprasad V, Spaner D, Verulkar S, Mandal NP, Sinha PK, Peeraju P, Dongre PR, Mahto RN, Atlin G (2009). Characterization of the effect of a QTL for drought resistance in rice, QTL12.1, over a range of environments in the Philippines and eastern India. Euphytica.

[CR7] Bernier J, Serraj R, Kumar A, Venuprasad R, Impa S, Gowda RPV, Oane R, Spaner D, Atlin G (2009). The large-effect drought-resistance QTL12.1 increases water uptake in upland rice. Field Crops Res.

[CR8] Bingham IJ, Stevenson EA (1993). Control of root growth: effects of carbohydrates on the extension branching and rate of respiration of different fractions of wheat roots. Physiol Plant.

[CR9] Bussis D, Heineke D (1998). Acclimation of potato plants to polyethylene glycol-induced water deficit—II. Contents and subcellular distribution of organic solutes. J Exp Bot.

[CR10] Chapman N, Miller AJ, Lindsey K, Whalley WR (2012). Roots, water and nutrient acquisition: let’s get physical. Trends Plant Sci.

[CR11] Chaves MM (1991). Effects of water deficits on carbon assimilation. J Exp Bot.

[CR12] Degenkolbe T, Do PT, Kopka J, Zuther E, Hinch DK, Kohl KI (2013). Identification of drought tolerance markers in a diverse population of rice cultivars by expression and metabolite profiling. PLoS ONE.

[CR13] deMendiburu, F (2014). Agricolae: Statistical Procedures for Agricultural Research. R package version 1.2-0. http://CRAN.R-project.org/package=agricolae

[CR14] Dixit S, Swamy BPM, Vikram P, Bernier T, Sta Cruz MT, Amante M, Atri D, Kumar A (2012). Increased drought tolerance and wider adaptability of qDTY 12.1 conferred by its interaction with qDTY 2.3 and qDTY 3.2. Mol breed.

[CR15] Drossopoulos JB, Karamanos AJ, Niavis A (1985). Changes in amino acid compounds during the development of two wheat cultivars subjected to different degrees of water stress. Ann Bot.

[CR16] Evers D, Lefevre I, Legay S, Lamoureux D, Hausman JF, Gutierrez Rosales RO, Tincopa Marca LR, Hoffmann L, Bonierbale M, Schafleitner R (2010). Identification of drought-responsive compounds in potato through a combined transcriptomic and targeted metabolite approach. J Exp Bot.

[CR17] Flexas J, Medrano H (2002). Drought inhibition of photosynthesis in C3 plants: stomatal and non-stomatal limitations revisited. Ann Bot.

[CR18] Freixes S, Thibaud MC, Tardieu F, Muller B (2002). Root elongation and branching is related to local hexose concentration in *Arabidopsis thaliana* seedlings. Plant Cell Envir.

[CR19] Fukai S, Cooper M (1995). Development of drought-resistant cultivars using physio-morphological traits in rice. Field Crops Res.

[CR20] Galvez DA, Landhäusser SM, Tyree MT (2011). Root carbon reserve dynamics in aspen seedlings: does simulated drought induce reserve limitation?. Tree Physiol.

[CR21] Garris AJ, Tai TH, Coburn J, Kresovich S, McCouch S (2005). Genetic structure and diversity in *Oryza sativa* L. Genet.

[CR22] Giani S, Brevario D (1996). Rice β- tubulin mRNA levels are modulated during flower development and is response to external stimuli. Plant Sci.

[CR23] Good AG, Johnson SJ, De Pauw M, Carroll RT, Savidov N, Vidmar J, Lu Z, Taylor G, Stroeher V (2007). Engineering nitrogen use efficiency with alanine aminotransferase. Can J Bot.

[CR24] Grayson M (2013). Agriculture and drought. Nature.

[CR25] Gupta AK, Kaur N (2005). Sugar signalling and gene expression in relation to carbohydrate metabolism under abiotic stresses in plants. J Biol Sci.

[CR26] Hasanuzzaman M, Nahar K, Alam MM, Roychowdhury R, Fujita M (2013). Physiological, biochemical, and molecular mechanisms of heat stress tolerance in plants. Int J Mol Sci.

[CR27] Henry A, Dixit S, Mandal NP, Anantha MS, Torres R, Kumar A (2014). Grain yield and physiological traits of rice lines with the drought yield QTL qDTY12.1 showed different responses to drought and soil characteristics in upland environments. Funct Plant Biol.

[CR28] Hien DT, Jacobs M, Agenon G, Hermans C, Thu TT, Son LV, Roosens NH (2003). Accumulation and d1-pyrroline-5-carboxylate synthetase gene properties in three rice cultivars differing in salinity and drought tolerance. Plant Sci.

[CR29] Hilal M, Parrado MF, Rosa M, Gallardo M, Orce L, Massa ED, Gonzalez JA, Prado FE (2004). Epidermal lignin deposition in quinoa cotyledons in response to UV-B radiation. Photochem Photobiol.

[CR30] Ho CL, Saito K (2001). Molecular biology of the plastidic phosphorylated serine biosynthetic pathway in Arabidopsis thaliana. Amino Acids.

[CR31] Ho SL, Chao YC, Tong WF, Yu SM (2001). Sugar coordinately and differentially regulates growth- and stress-related gene expression via a complex signal transduction network and multiple control mechanisms. Plant Physiol.

[CR32] Hussey PJ, Ketelaar T, Deeks MJ (2006). Control of the actin cytoskeleton in plant cell growth. Ann Rev Plant Biol.

[CR33] Hwang JU, Suh S, Yi H, Kim J, Lee Y (1997). Actin filaments modulate both stomatal opening and inward K^+^-channel activities in guard cells of *Vicia faba* L. Plant Physiol.

[CR34] Jagadish SVK, Septiningsih EM, Kohli A, Thomson MJ, Ye C, Redoña E, Kumar A, Gregorio GB, Wassmann R, Ismail AM, Singh RK (2012). Genetic advances in adapting rice to a rapidly changing climate. J Agron Crop Sci.

[CR35] Jang JC, Sheen J (1997). Sugar sensing in higher plants. Plant Cell.

[CR36] Kant S, Kant P, Raveh E, Barak S (2006). Evidence that differential gene expression between the halophyte, *Thellungiella halophila,* and *Arabidopsis thaliana* is responsible for higher levels of the compatible osmolyte proline and tight control of Na+ uptake in *T. halophila*. Plant Cell Environ.

[CR37] Khush GS (1997). Origin, dispersal, cultivation and variation of rice. Plant Mol Biol.

[CR38] Kichey T, Hirel B, Heumez E, Dubois F, Gouis JL (2007). In winter wheat (*Triticum aestivum* L.), post-anthesis nitrogen uptake and remobilisation to the grain correlates with agronomic traits and nitrogen physiological markers. Field Crop Res.

[CR39] Kim M, Hepler PK, Eun SO, Ha KS, Lee Y (1995). Actin filaments in mature guard cells are radially distributed and involved in stomatal movement. Plant Physiol.

[CR40] Kishor PBK, Sreenivasulu N (2013). Is proline accumulation *per se* correlated with stress tolerance or is proline homeostasis a more critical issue?. Plant Cell Environ.

[CR41] Kohli A, Narciso JO, Miro B, Raorane M (2012). Root proteases: reinforced links between nitrogen uptake and mobilization and drought tolerance. Physiol Plant.

[CR42] Kumar A, Dixit S, Ram T, Yadaw RB, Mishra KK, Mandal NP (2014). Breeding high-yielding drought tolerant rice: genetic variations and conventional molecular approaches. J Exp Bot Adv Access.

[CR43] Lauteri M, Haworth M, Serraj R, Monteverdi MC, Centritto M (2014). Photosynthetic diffusional constraints affect yield in drought stressed rice cultivars during flowering. PLoS ONE.

[CR44] Lea PJ, Ireland RJ, Singh BK (1999). Nitrogen metabolism in higher plants. Plant amino acids.

[CR45] Lemichez E, Wu Y, Sanchez JP, Mettouchi A, Mathur J, Chua NH (2001). Inactivation of *AtRac1* by abscisic acid is essential for stomatal closure. Genes Dev.

[CR46] Limami AM, Glévarec G, Ricoult C, Cliquet JB, Planchet E (2008). Concerted modulation of alanine and glutamate metabolism in young *Medicago truncatula* seedlings under hypoxic stress. J Exp Bot.

[CR47] Lin BB (2011). Resilience in agriculture through crop diversification: adaptive management for environmental change. Bioscience.

[CR48] Mackill DJ, Coffman WR, Garrity DP (1996). Rain-fed lowland rice improvement.

[CR49] Masclaux-Daubresse C, Reisdorf-Cren M, Orsel M (2008). Leaf nitrogen remobilization for plant development and grain filling. Plant Biol.

[CR50] Mir RR, Zaman-Allah M, Sreenivasulu N, Trethowan R, Varshney RK (2012). Integrated genomics, physiology and breeding approaches for improving drought tolerance in crops. Theorand App Gen.

[CR51] Mishra KK, Vikram P, Yadaw RB, Swamy BPM, Dixit S, Sta Cruz MT, Maturan P, Marker S, Kumar A (2013). qDTY_12.1_: a locus with a consistent effect on grain yield under drought in rice. BMC Genet.

[CR52] Miyashita Y, Dolferus R, Ismind KP, Good AG (2007). Alanine aminotransferase catalyses the breakdown of alanine after hypoxia in *Arabidopsis thaliana*. Plant J.

[CR53] Novitskaya L, Trevanion SJ, Driscoll S, Foyer CH, Noctor G (2002). How does photorespiration modulate leaf amino acid contents? A dual approach through modelling and metabolite analysis. Plant, Cell Environ.

[CR54] Oliver SN, Tiessen A, Fernie AR, Geigenberger P (2008). Decreased expression of plastidial adenylate kinase in potato tubers results in an enhanced rate of respiration and a stimulation of starch synthesis that is attributable to post-translational redox-activation of ADP-glucose pyrophosphorylase. J Exp Bot.

[CR55] O’Toole JC, International Rice Research Institute (1982). Adaptation of rice to drought prone environments. Drought resistance in crops with emphasis on rice.

[CR56] Pinheiro C, Chaves MM (2011). Photosynthesis and drought: can we make metabolic connections from available data?. J Exp Bot.

[CR57] Ramanjulu S, Sudhakar C (1997). Drought tolerance is partly related to amino acid accumulation and ammonia assimilation: a comparative study in two mulberry genotypes differing in drought sensitivity. J Plant Physiol.

[CR58] Raorane ML, Pabuayon IM, Varadarajan AR, Mutte SK, Kumar A, Treumann A, Kohli A (2015) Proteomic insight into the role of the large-effect QTL *qDTY*_*12.1*_ for rice yield under drought. Molecular Breeding (this issue)

[CR59] Rocha M, Sodek L, Licausi F, Hameed MW, Dornelas MC, Dongen JT (2010). Analysis of alanine aminotransferase in various organs of soybean (*Glycine max*) and independence of different nitrogen fertilisers during hypoxic stress. Amino Acids.

[CR60] Schatz MC, Maron LG, Stein JC, Wences AH, Gurtowski J, Biggers E, Lee H, Kramer M, Antoniou E, Ghiban E, Wright MH, Chia J, Ware D, McCouch SR, McCombie WR (2014). Whole genome *de novo* assemblies of three divergent strains of rice, *Oryza sativa,* document novel gene space of aus and indica. Genome Biol.

[CR61] Serraj R, McNally KL, Slamet-Loedin I, Kohli A, Haefele SM, Atlin G, Kumar A (2011). Drought resistance improvement in rice: an integrated genetic and resource management strategy. Plant Prod Sci.

[CR62] Sharma S, Villamor JG, Verslues PE (2011). Essential role of tissue-specific proline synthesis and catabolism in growth and redox balance at low water potential. Plant Physiol.

[CR63] Sheoran IS, Koonjul P, Attieh J, Saini HS (2014). Water-stress-induced inhibition of alpha-tubulin gene expression during growth, and its implications for reproductive success in rice. Plant Physiol Biochem.

[CR64] Sicher RC, Timlin D, Bailey B (2012). Responses of growth and primary metabolism of water-stressed barley roots to rehydration. J Plant Physiol.

[CR65] Silvente S, Sobolev AP, Lara M (2012). Metabolite adjustments in drought tolerant and sensitive soybean genotypes in response to water stress. PLoS ONE.

[CR66] Smith AM, Denyer K, Martin C (1997). The synthesis of the starch granule. Ann Rev Plant Physiol Plant Mol Biol.

[CR67] Stewart CR, Hanson AD, Turner NC, Kramer PJ (1980). Proline accumulation as a metabolic response to water stress. Adaptation of plants to water and high temperature stress.

[CR68] Suralta RR (2010). Plastic root system development responses to drought-enhanced nitrogen uptake during progressive soil drying conditions in rice. Philipp Agric Sci.

[CR69] Swamy BPM, Vikram P, Dixit S, Ahmed HU, Kumar A (2011). Meta-analysis of grain yield QTL identified during agricultural drought in grasses showed consensus. BMC Genom.

[CR70] Swarbreck SM, Defoin-Platel M, Hindle M, Saqi M, Habash DZ (2011). New perspectives on glutamine synthetase in grasses. J Exp Botany.

[CR71] Sweetlove LJ, Beard KFM, Nunes-Nesi A, Fernie AR, Ratcliffe RG (2010). Not just a circle: flux modes in the plant TCA cycle. Trends Plant Sci.

[CR72] Szabados L, Savouré A (2010). Proline: a multifunctional amino acid. Trends Plant Sci.

[CR73] R Core Team (2013) R: A language and environment for statistical computing. R Foundation for Statistical Computing, Vienna, Austria. ISBN 3-900051-07-0, http://www.R-project.org/

[CR74] Thavarajah D, Ball RS (2006). Drought-induced changes in free amino acid and ureide concentrations of nitrogen-fixing chickpea. Can J Plant Sci.

[CR75] Tran LS, Nakashima K, Shinozaki K, Yamaguchi-Shinozaki K (2007). Plant gene networks in osmotic stress response: from genes to regulatory networks. Methods Enzymol.

[CR76] Ufaz S, Galili G (2008). Improving the content of essential amino acids in crop plants: goals and opportunities. Plant Physiol.

[CR77] Verslues PE, Sharma S (2010). Proline metabolism and its implications for plant-environment interaction. Arabidopsis Book.

[CR78] Verslues PE, Sharp RE (1999). Proline accumulation in maize (Zea mays L.) primary roots at low water potentials. II. Metabolic source of increased proline deposition in the elongation zone. Plant Physiol.

[CR79] Waditee R, Bhuiyan NH, Hirata E, Hibino T, Tanaka Y, Shikata M, Takabe T (2007). Metabolic engineering for betaine accumulation in microbes and plants. J Biol Chem.

[CR80] Weidner S, Karolak M, Karamac M, Kosinska A, Amarowicz R (2009). Phenolic compounds and properties of antioxidants in grapevine roots (*Vitis Vinifera* L.) under drought stress followed by recovery. Acta Soc Bot Pol.

[CR81] Zhu J, Kaeppler SM, Lynch JP (2005). Mapping of QTL controlling root hair in maize (*Zea mays* L.) under phosphorous deficiency. Plant Soil.

