# Supplementary material for: Variation in primary metabolites in parental and near-isogenic lines of the QTL qDTY12.1: altered roots and flag leaves but similar spikelets of rice under drought
Source: Mol Breed. 2015 Jun 2;35(6):138. doi: 10.1007/s11032-015-0322-5 (PMC4451464; doi:10.1007/s11032-015-0322-5)
Supplement: Supplementary file 1 — Supplementary material 1 (PDF 570 kb) [file 11032_2015_322_MOESM1_ESM.pdf]

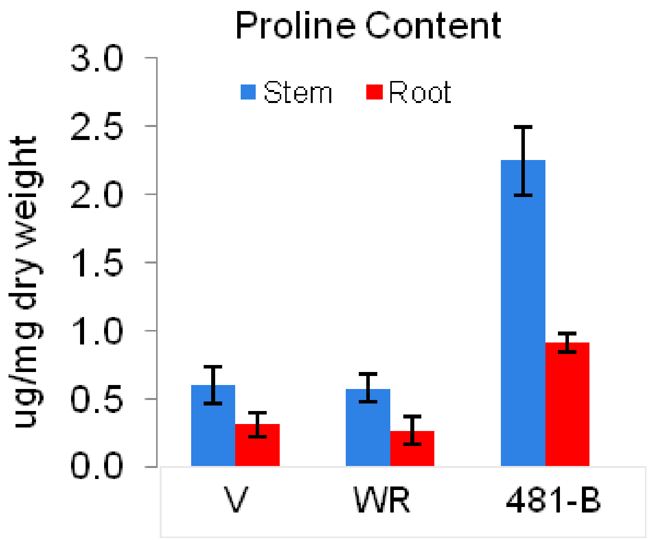


**Supplementary Figure 1. Proline content estimation:** Amount of proline measured in micrograms per milligram of tissue dry weight in the stem and root of the three genotypes under drought revealed greatest amount in the 481-B stem and root. This data suggested movement of proline from the flag leaf to the root.

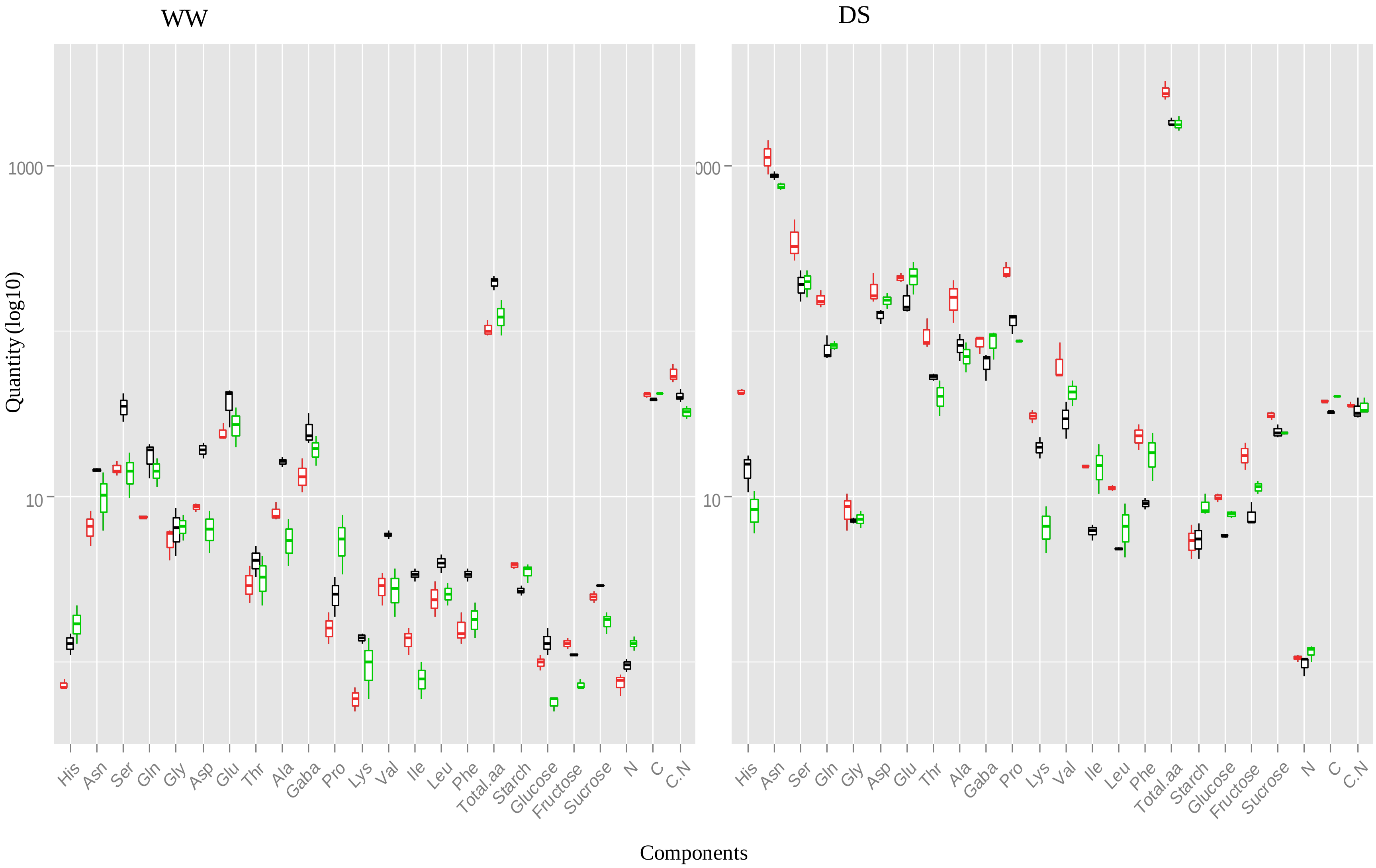


**Supplementary Figure 1: Boxplots for metabolite contents in roots.** Boxplots represent all metabolites grouped by genotype. The left and the right panels represent well-watered (WW) and drought stress (DS) conditions. Metabolites are represented on the horizontal axis and quantities on the vertical axis. The data is represented on a log10 scale to accommodate different unit magnitudes in one representation. The horizontal white lines represent the values 1, 10, 100 and 1000. Each vertical white line represents a metabolite, and the 3 boxes closest to it are the 3 genotypes: Vandana on the white line is coded in black, 481-B on the left of the line is in red, and Way Rarem on the right is in green. In each box, the horizontal line represents the median of the data, the upper quartile represents the 75th percentiles and the lower quartile represents the 25th percentile. The whiskers represented the 95% and 5% of the data and the circles, when present, signified the outliers. The amino acids were measured in pmol/mg; starch, glucose, fructose and sucrose in μmol/gr fresh weight; and N and C in %, where C/N is their ratio.

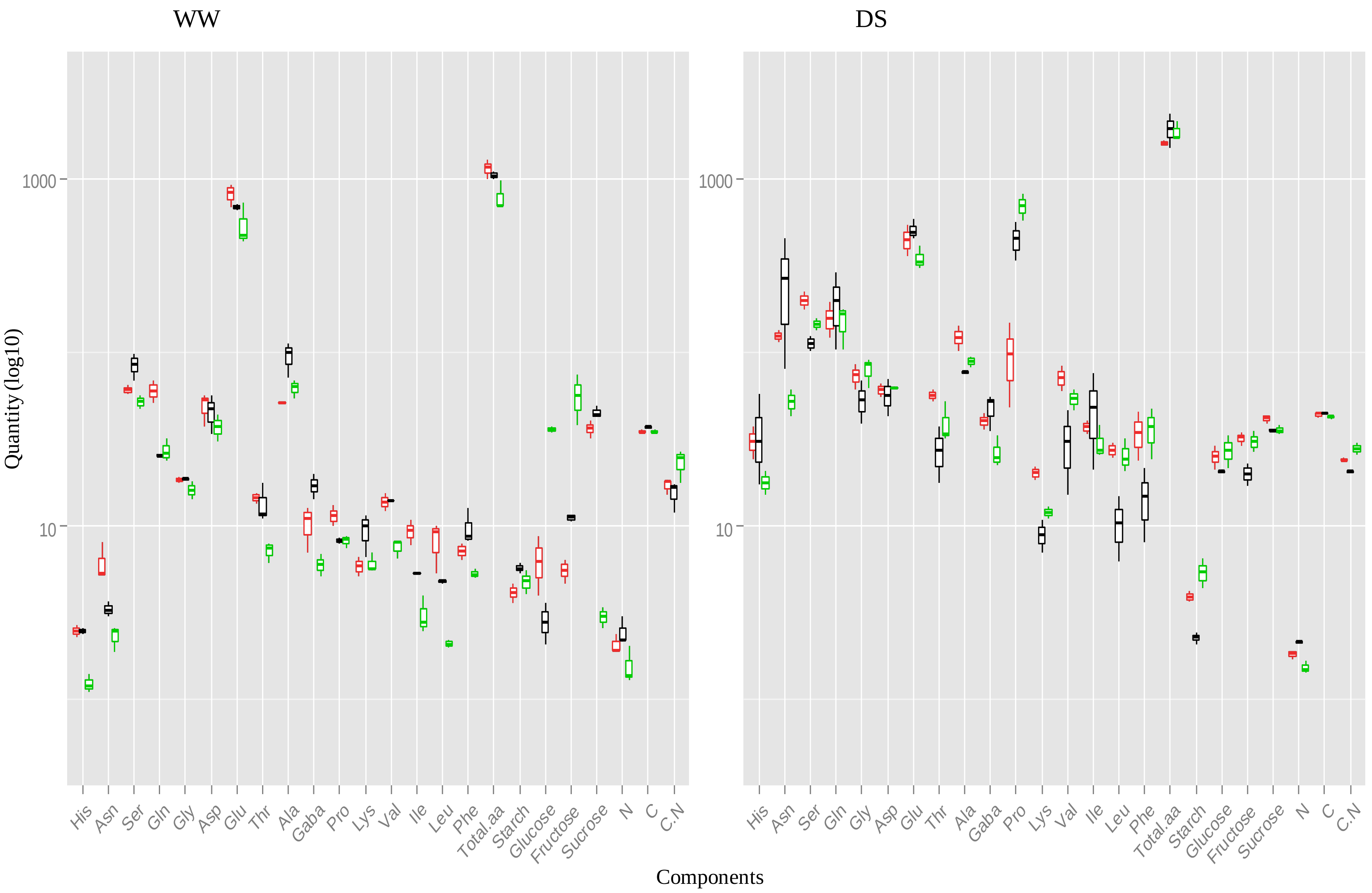


**Supplementary Figure 2: Boxplots for metabolite contents in flag leaves.** Boxplots represent all metabolites grouped by genotype. The left and the right panels represent well-watered (WW) and drought stress (DS) conditions. Metabolites are represented on the horizontal axis and quantities on the vertical axis. The data is represented on a log10 scale to accommodate different unit magnitudes in one representation. The horizontal white lines represent the values 1, 10, 100 and 1000. Each vertical white line represents a metabolite, and the 3 boxes closest to it are the 3 genotypes: Vandana on the white line is coded in black, 481-B on the left of the line is in red, and Way Rarem on the right is in green. In each box, the horizontal line represents the median of the data, the upper quartile represents the 75th percentiles and the lower quartile represents the 25th percentile. The whiskers represented the 95% and 5% of the data and the circles, when present, signified the outliers. The amino acids were measured in pmol/mg; starch, glucose, fructose and sucrose in μmol/gr fresh weight; and N and C in %, where C/N is their ratio.

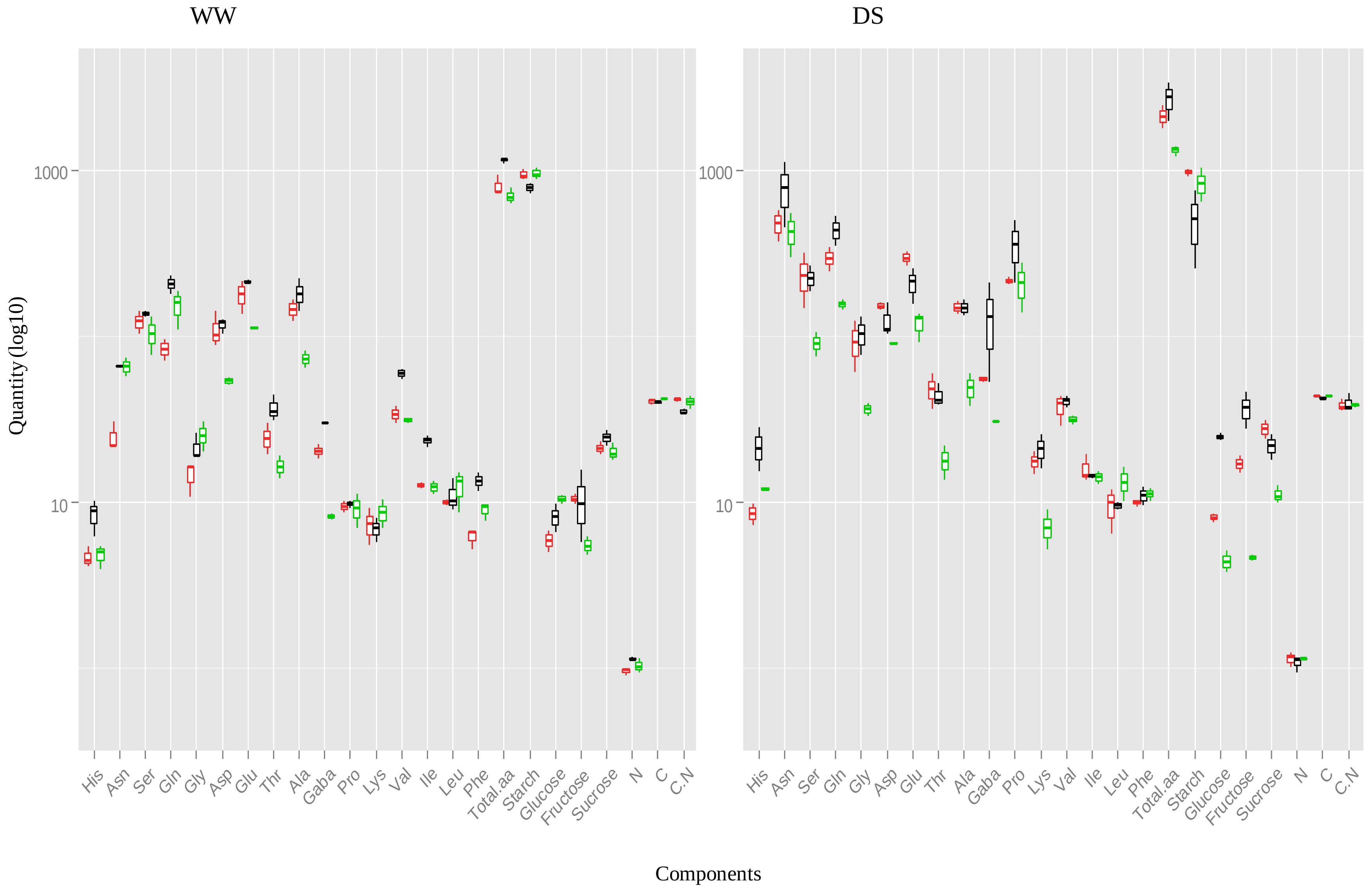


**Supplementary Figure 3: Boxplots for metabolite contents in spikelets.** Boxplots represent all metabolites grouped by genotype. The left and the right panels represent well-watered (WW) and drought stress (DS) conditions. Metabolites are represented on the horizontal axis and quantities on the vertical axis. The data is represented on a log10 scale to accommodate different unit magnitudes in one representation. The horizontal white lines represent the values 1, 10, 100 and 1000. Each vertical white line represents a metabolite, and the 3 boxes closest to it are the 3 genotypes: Vandana on the white line is coded in black, 481-B on the left of the line is in red, and Way Rarem on the right is in green. In each box, the horizontal line represents the median of the data, the upper quartile represents the 75th percentiles and the lower quartile represents the 25th percentile. The whiskers represented the 95% and 5% of the data and the circles, when present, signified the outliers. The amino acids were measured in pmol/mg; starch, glucose, fructose and sucrose in μmol/gr fresh weight; and N and C in %, where C/N is their ratio.

# Comparison of variation in primary metabolites between the parental and near-isogenic lines of the rice QTL *qDTY*$_{12.1}$ for yield under drought: changes in roots and flag leaves help maintain spikelet characters

Molecular Breeding

Manish L Raorane[1], Isaiah M Pabuayon[1], Berta Miro[1], Rajesh Kalladan[2#], Mohammad Reza-Hazirezai[2], Rowena H Oane[1], Arvind Kumar[1], Nese Sreenivasulu[1, 2], Amelia Henry[1], Ajay Kohli[1*].

***Correspondence:**

Ajay Kohli: a.kohli@irri.org

Tel: +6325805600. Extn. 2764

## Supplementary Information

**Supplementary Table 1: One-way ANOVA outputs for each metabolite in roots, flag leaves and spikelets.** Each table contains the output values for each metabolite in root tissue. The table describes the treatment significance (T), the genotype significance (G), the genotype x treatment (GxE) interaction significance and Res for the residuals. The first column represents the degrees of freedom, the second the sum of squares, the third the mean of the sum of squares, the fourth one is the F test and the last is the p for the F test.

### Flag Leaf

His

| | Df | Sum Sq | Mean Sq | F value | Pr(>F) |
|---|---|---|---|---|---|
| T | 1 | 3031.6 | 3031.6 | 38.407 | 4.61E-05 |
| G | 2 | 288 | 144 | 1.824 | 0.203 |
| GxE | 2 | 210.7 | 105.4 | 1.335 | 0.3 |
| Res | 12 | 947.2 | 78.9 | | |

Gly

| | Df | Sum Sq | Mean Sq | F value | Pr(>F) |
|---|---|---|---|---|---|
| T | 1 | 11817 | 11817 | 120.895 | 1.27E-07 |
| G | 2 | 486 | 243 | 2.487 | 0.1248 |
| GxE | 2 | 655 | 328 | 3.351 | 0.0698 |
| Res | 12 | 1173 | 98 | | |

Val

| | Df | Sum Sq | Mean Sq | F value | Pr(>F) |
|---|---|---|---|---|---|
| T | 1 | 7288 | 7288 | 101.697 | 3.27E-07 |
| G | 2 | 1240 | 620 | 8.649 | 0.00472 |
| GxE | 2 | 1342 | 671 | 9.362 | 0.00355 |
| Res | 12 | 860 | 72 | | |

Ala

| | Df | Sum Sq | Mean Sq | F value | Pr(>F) |
|---|---|---|---|---|---|
| T | 1 | 3110 | 3110 | 20.006 | 0.000762 |
| G | 2 | 483 | 241.7 | 1.555 | 0.250877 |
| GxE | 2 | 5896 | 2948.2 | 18.965 | 0.000193 |
| Res | 12 | 1865 | 155.5 | | |

Pro

| | Df | Sum Sq | Mean Sq | F value | Pr(>F) |
|---|---|---|---|---|---|
| T | 1 | 748313 | 748313 | 148.22 | 4.12E-08 |
| G | 2 | 273041 | 136520 | 27.04 | 3.59E-05 |
| GxE | 2 | 279414 | 139707 | 27.67 | 3.20E-05 |
| Res | 12 | 60583 | 5049 | | |

Lys

| | Df | Sum Sq | Mean Sq | F value | Pr(>F) |
|---|---|---|---|---|---|
| T | 1 | 194.04 | 194.04 | 77.64 | 1.38E-06 |
| G | 2 | 61.94 | 30.97 | 12.39 | 0.00121 |
| GxE | 2 | 165.28 | 82.64 | 33.06 | 1.31E-05 |
| Res | 12 | 29.99 | 2.5 | | |

Ser

| | Df | Sum Sq | Mean Sq | F value | Pr(>F) |
|---|---|---|---|---|---|
| T | 1 | 34357 | 34357 | 189.85 | 1.02E-08 |
| G | 2 | 3986 | 1993 | 11.01 | 0.00192 |
| GxE | 2 | 9246 | 4623 | 25.55 | 4.74E-05 |
| Res | 12 | 2172 | 181 | | |

Thr

| | Df | Sum Sq | Mean Sq | F value | Pr(>F) |
|---|---|---|---|---|---|
| T | 1 | 3854 | 3854 | 91.981 | 5.61E-07 |
| G | 2 | 759 | 380 | 9.058 | 0.004 |
| GxE | 2 | 600 | 300 | 7.158 | 0.00899 |
| Res | 12 | 503 | 42 | | |

Glu

| | Df | Sum Sq | Mean Sq | F value | Pr(>F) |
|---|---|---|---|---|---|
| T | 1 | 277140 | 277140 | 28.203 | 0.000185 |
| G | 2 | 119081 | 59541 | 6.059 | 0.015171 |
| GxE | 2 | 33828 | 16914 | 1.721 | 0.220183 |
| Res | 12 | 117918 | 9827 | | |

Asp

| | Df | Sum Sq | Mean Sq | F value | Pr(>F) |
|---|---|---|---|---|---|
| T | 1 | 1156.8 | 1156.8 | 14.134 | 0.00272 |
| G | 2 | 81.1 | 40.5 | 0.495 | 0.62134 |
| GxE | 2 | 198.3 | 99.2 | 1.212 | 0.3317 |
| Res | 12 | 982.1 | 81.8 | | |

Ile

| | Df | Sum Sq | Mean Sq | F value | Pr(>F) |
|---|---|---|---|---|---|
| T | 1 | 4812 | 4812 | 36.282 | 6.00E-05 |
| G | 2 | 316 | 158 | 1.191 | 0.337 |
| GxE | 2 | 242 | 121 | 0.913 | 0.428 |

Leu

| | Df | Sum Sq | Mean Sq | F value | Pr(>F) |
|---|---|---|---|---|---|
| T | 1 | 1181 | 1181 | 108.61 | 2.29E-07 |
| G | 2 | 317.1 | 158.6 | 14.58 | 0.000614 |
| GxE | 2 | 258.4 | 129.2 | 11.88 | 0.001428 |

| | | | |
|---|---|---|---|
| Res | 12 | 1591 | 133 |

| | | | |
|---|---|---|---|
| Res | 12 | 130.5 | 10.9 |

Gln

| | Df | Sum Sq | Mean Sq | F value | Pr(>F) |
|---|---|---|---|---|---|
| T | 1 | 34357 | 34357 | 189.85 | 1.02E-08 |
| G | 2 | 3986 | 1993 | 11.01 | 0.00192 |
| GxE | 2 | 9246 | 4623 | 25.55 | 4.74E-05 |
| Res | 12 | 2172 | 181 | | |

Asn

| | Df | Sum Sq | Mean Sq | F value | Pr(>F) |
|---|---|---|---|---|---|
| T | 1 | 93283 | 93283 | 16.172 | 0.0017 |
| G | 2 | 35655 | 17827 | 3.091 | 0.0827 |
| GxE | 2 | 35474 | 17737 | 3.075 | 0.0835 |
| Res | 12 | 69220 | 5768 | | |

Gaba

| | Df | Sum Sq | Mean Sq | F value | Pr(>F) |
|---|---|---|---|---|---|
| T | 1 | 3278 | 3278 | 107.393 | 2.43E-07 |
| G | 2 | 766 | 383 | 12.555 | 0.00114 |
| GxE | 2 | 88 | 44 | 1.445 | 0.27388 |
| Res | 12 | 366 | 31 | | |

Phe

| | Df | Sum Sq | Mean Sq | F value | Pr(>F) |
|---|---|---|---|---|---|
| T | 1 | 2005.6 | 2005.6 | 39.964 | 3.82E-05 |
| G | 2 | 287.4 | 143.7 | 2.863 | 0.0962 |
| GxE | 2 | 595.3 | 297.6 | 5.931 | 0.0162 |
| Res | 12 | 602.2 | 50.2 | | |

Total aa

| | Df | Sum S | q Mean S | q F valu | Pr(>F) |
|---|---|---|---|---|---|
| T | 1 | 2982415 | 2982415 | 59.503 | 5.45E-06 |
| G | 2 | 91218 | 45609 | 0.91 | 0.4286 |
| GxE | 2 | 313046 | 156523 | 3.123 | 0.0809 |
| Res | 12 | 601462 | 50122 | | |

Starch

| | Df | Sum Sq | Mean Sq | F value | Pr(>F) |
|---|---|---|---|---|---|
| T | 1 | 4.621 | 4.621 | 12.5 | 0.004108 |
| G | 2 | 4.866 | 2.433 | 6.58 | 0.01177 |
| GxE | 2 | 13.852 | 6.926 | 18.73 | 0.000204 |
| Res | 12 | 4.437 | 0.37 | | |

Glucose

| | Df | Sum Sq | Mean Sq | F value | Pr(>F) |
|---|---|---|---|---|---|
| T | 1 | 401.4 | 401.4 | 43.58 | 2.53E-05 |
| G | 2 | 1325.7 | 662.8 | 71.97 | 2.08E-07 |
| GxE | 2 | 709.1 | 354.6 | 38.5 | 6.01E-06 |
| Res | 12 | 110.5 | 9.2 | | |

Fructose

| | Df | Sum Sq | Mean Sq | F value | Pr(>F) |
|---|---|---|---|---|---|
| T | 1 | 47 | 47 | 0.755 | 0.401926 |
| G | 2 | 2815.7 | 1407.8 | 22.595 | 8.53E-05 |
| GxE | 2 | 2091.7 | 1045.8 | 16.785 | 0.000333 |
| Res | 12 | 747.7 | 62.3 | | |

Sucrose

| | Df | Sum Sq | Mean Sq | F value | Pr(>F) |
|---|---|---|---|---|---|
| T | 1 | 407.1 | 407.1 | 59.79 | 5.32E-06 |
| G | 2 | 1630.5 | 815.3 | 119.75 | 1.18E-08 |
| GxE | 2 | 1402.7 | 701.4 | 103.02 | 2.78E-08 |
| Res | 12 | 81.7 | 6.8 | | |

N

| | Df | Sum Sq | Mean Sq | F value | Pr(>F) |
|---|---|---|---|---|---|
| T | 1 | 0.1824 | 0.1824 | 2.149 | 0.16839 |
| G | 2 | 1.7539 | 0.877 | 10.333 | 0.00246 |
| GxE | 2 | 0.0664 | 0.0332 | 0.391 | 0.68469 |
| Res | 12 | 1.0184 | 0.0849 | | |

C

| | Df | Sum Sq | Mean Sq | F value | Pr(>F) |
|---|---|---|---|---|---|
| T | 1 | 269.11 | 269.11 | 408.291 | 1.24E-10 |
| G | 2 | 12.58 | 6.29 | 9.545 | 0.00331 |
| GxE | 2 | 1.49 | 0.74 | 1.128 | 0.35559 |
| Res | 12 | 7.91 | 0.66 | | |

C/N

| | Df | Sum Sq | Mean Sq | F value | Pr(>F) |
|---|---|---|---|---|---|
| T | 1 | 140.89 | 140.89 | 21.383 | 0.000586 |
| G | 2 | 173.51 | 86.75 | 13.167 | 0.000941 |
| GxE | 2 | 3.89 | 1.94 | 0.295 | 0.749713 |
| Res | 12 | 79.06 | 6.59 | | |

## 1 Roots

His

| | Df | Sum Sq | Mean Sq | F value | Pr(>F) |
|---|---|---|---|---|---|
| T | 1 | 1949 | 1949 | 522.8 | 2.91E-11 |
| G | 2 | 966.3 | 483.1 | 129.6 | 7.50E-09 |
| GxE | 2 | 1073 | 536.5 | 143.9 | 4.11E-09 |
| Res | 12 | 44.7 | 3.7 | | |

Gly

| | Df | Sum Sq | Mean Sq | F value | Pr(>F) |
|---|---|---|---|---|---|
| T | 1 | 9.68 | 9.68 | 4.668 | 0.0517 |
| G | 2 | 0.058 | 0.029 | 0.014 | 0.9862 |
| GxE | 2 | 5.293 | 2.647 | 1.276 | 0.3144 |
| Res | 12 | 24.887 | 2.074 | | |

Ala

| | Df | Sum Sq | Mean Sq | F value | Pr(>F) |
|---|---|---|---|---|---|
| T | 1 | 39640 | 39640 | 93.98 | 5.00E-07 |
| G | 2 | 6731 | 3366 | 7.98 | 0.00625 |
| GxE | 2 | 7330 | 3665 | 8.69 | 0.00464 |
| Res | 12 | 5061 | 422 | | |

Val

| | Df | Sum Sq | Mean Sq | F value | Pr(>F) |
|---|---|---|---|---|---|
| T | 1 | 7846 | 7846 | 109.491 | 2.19E-07 |
| G | 2 | 779 | 390 | 5.438 | 0.02083 |
| GxE | 2 | 1067 | 534 | 7.447 | 0.00789 |
| Res | 12 | 860 | 72 | | |

Asp

| | Df | Sum Sq | Mean Sq | F value | Pr(>F) |
|---|---|---|---|---|---|
| T | 1 | 89973 | 89973 | 268.834 | 1.40E-09 |
| G | 2 | 1612 | 806 | 2.409 | 0.132 |
| GxE | 2 | 3372 | 1686 | 5.038 | 0.0258 |
| Res | 12 | 4016 | 335 | | |

Glu

| | Df | Sum Sq | Mean Sq | F value | Pr(>F) |
|---|---|---|---|---|---|
| T | 1 | 121393 | 121393 | 189.518 | 1.03E-08 |
| G | 2 | 2265 | 1133 | 1.768 | 0.2123 |
| GxE | 2 | 4954 | 2477 | 3.867 | 0.0506 |
| Res | 12 | 7686 | 641 | | |

Lys

| | Df | Sum Sq | Mean Sq | F value | Pr(>F) |
|---|---|---|---|---|---|
| T | 1 | 1483.3 | 1483.3 | 432.38 | 8.85E-11 |
| G | 2 | 424.6 | 212.3 | 61.88 | 4.77E-07 |
| GxE | 2 | 450.6 | 225.3 | 65.67 | 3.44E-07 |
| Res | 12 | 41.2 | 3.4 | | |

Pro

| | Df | Sum Sq | Mean Sq | F value | Pr(>F) |
|---|---|---|---|---|---|
| T | 1 | 89747 | 89747 | 518.39 | 3.06E-11 |
| G | 2 | 16722 | 8361 | 48.29 | 1.82E-06 |
| GxE | 2 | 18237 | 9119 | 52.67 | 1.14E-06 |
| Res | 12 | 2078 | 173 | | |

Ser

| | Df | Sum Sq | Mean Sq | F value | Pr(>F) |
|---|---|---|---|---|---|
| T | 1 | 233085 | 233085 | 98.581 | 3.87E-07 |
| G | 2 | 22819 | 11409 | 4.826 | 0.029 |
| GxE | 2 | 29566 | 14783 | 6.252 | 0.0138 |
| Res | 12 | 28373 | 2364 | | |

Thr

| | Df | Sum Sq | Mean Sq | F value | Pr(>F) |
|---|---|---|---|---|---|
| T | 1 | 16080 | 16080 | 169.29 | 1.95E-08 |
| G | 2 | 2453 | 1226 | 12.91 | 0.00102 |
| GxE | 2 | 2562 | 1281 | 13.49 | 0.000852 |
| Res | 12 | 1140 | 95 | | |

Gln

| | Df | Sum Sq | Mean Sq | F value | Pr(>F) |
|---|---|---|---|---|---|
| T | 1 | 38411 | 38411 | 359.23 | 2.61E-10 |
| G | 2 | 4530 | 2265 | 21.18 | 0.000116 |
| GxE | 2 | 7119 | 3559 | 33.29 | 1.27E-05 |
| Res | 12 | 1283 | 107 | | |

Asn

| | Df | Sum Sq | Mean Sq | F value | Pr(>F) |
|---|---|---|---|---|---|
| T | 1 | 3736922 | 3736922 | 310.566 | 6.08E-10 |
| G | 2 | 119207 | 59603 | 4.953 | 0.027 |
| GxE | 2 | 125267 | 62634 | 5.205 | 0.0236 |
| Res | 12 | 144391 | 12033 | | |

*Rice primary metabolites variation under drought*

Leu

| | Df | Sum Sq | Mean Sq | F value | Pr(>F) |
|---|---|---|---|---|---|
| T | 1 | 93.85 | 93.85 | 85.97 | 8.06E-07 |
| G | 2 | 22.52 | 11.26 | 10.32 | 0.00247 |
| GxE | 2 | 48.64 | 24.32 | 22.28 | 9.12E-05 |
| Res | 12 | 13.1 | 1.09 | | |

Ile

| | Df | Sum Sq | Mean Sq | F value | Pr(>F) |
|---|---|---|---|---|---|
| T | 1 | 490.9 | 490.9 | 110.353 | 2.10E-07 |
| G | 2 | 47.4 | 23.7 | 5.333 | 0.022019 |
| GxE | 2 | 133.2 | 66.6 | 14.967 | 0.000549 |
| Res | 12 | 53.4 | 4.4 | | |

Phe

| | Df | Sum Sq | Mean Sq | F value | Pr(>F) |
|---|---|---|---|---|---|
| T | 1 | 966.5 | 966.5 | 106.97 | 2.49E-07 |
| G | 2 | 117.7 | 58.8 | 6.512 | 0.01216 |
| GxE | 2 | 200.2 | 100.1 | 11.079 | 0.00188 |
| Res | 12 | 108.4 | 9 | | |

Gaba

| | Df | Sum Sq | Mean Sq | F value | Pr(>F) |
|---|---|---|---|---|---|
| T | 1 | 15506 | 15506 | 161.02 | 2.59E-08 |
| G | 2 | 210 | 105 | 1.09 | 0.3674 |
| GxE | 2 | 974 | 487 | 5.06 | 0.0255 |
| Res | 12 | 1156 | 96 | | |

Total aa

| | Df | Sum Sq | Mean Sq | F value | Pr(>F) |
|---|---|---|---|---|---|
| T | 1 | 18041023 | 18041023 | 585.07 | 1.50E-11 |
| G | 2 | 911074 | 455537 | 14.77 | 0.000581 |
| GxE | 2 | 1133757 | 566879 | 18.38 | 0.000222 |
| Res | 12 | 370030 | 30836 | 36 | |

Starch

| | Df | Sum Sq | Mean Sq | F value | Pr(>F) |
|---|---|---|---|---|---|
| T | 1 | 47.86 | 47.86 | 53.605 | 9.20E-06 |
| G | 2 | 13.99 | 7 | 7.837 | 0.00665 |
| GxE | 2 | 11.07 | 5.54 | 6.201 | 0.01415 |
| Res | 12 | 10.71 | 0.89 | | |

Glucose

| | Df | Sum Sq | Mean Sq | F value | Pr(>F) |
|---|---|---|---|---|---|
| T | 1 | 212.87 | 212.87 | 2152.59 | 6.56E-15 |
| G | 2 | 10.77 | 5.39 | 54.48 | 9.54E-07 |
| GxE | 2 | 14.92 | 7.46 | 75.44 | 1.60E-07 |
| Res | 12 | 1.19 | 0.1 | | |

Fructose

| | Df | Sum Sq | Mean Sq | F value | Pr(>F) |
|---|---|---|---|---|---|
| T | 1 | 574.6 | 574.6 | 260.33 | 1.68E-09 |
| G | 2 | 82.4 | 41.2 | 18.67 | 0.000207 |
| GxE | 2 | 73.8 | 36.9 | 16.72 | 0.000339 |
| Res | 12 | 26.5 | 2.2 | | |

Sucrose

| | Df | Sum Sq | Mean Sq | F value | Pr(>F) |
|---|---|---|---|---|---|
| T | 1 | 2642.6 | 2642.6 | 1780.22 | 2.04E-14 |
| G | 2 | 46.7 | 23.3 | 15.72 | 0.000445 |
| GxE | 2 | 40.6 | 20.3 | 13.68 | 0.000802 |
| Res | 12 | 17.8 | 1.5 | | |

N

| | Df | Sum Sq | Mean Sq | F value | Pr(>F) |
|---|---|---|---|---|---|
| T | 1 | 0.0154 | 0.01542 | 1.301 | 0.276325 |
| G | 2 | 0.3499 | 0.17493 | 14.756 | 0.000584 |
| GxE | 2 | 0.1585 | 0.07924 | 6.684 | 0.011203 |
| Res | 12 | 0.1423 | 0.01186 | | |

C

| | Df | Sum Sq | Mean Sq | F value | Pr(>F) |
|---|---|---|---|---|---|
| T | 1 | 63.89 | 63.89 | 125.93 | 1.02E-07 |
| G | 2 | 92.93 | 46.46 | 91.58 | 5.40E-08 |
| GxE | 2 | 16.73 | 8.37 | 16.49 | 0.000361 |
| Res | 12 | 6.09 | 0.51 | | |

C/N

| | Df | Sum Sq | Mean Sq | F value | Pr(>F) |
|---|---|---|---|---|---|
| T | 1 | 284.9 | 284.91 | 15.64 | 0.00191 |
| G | 2 | 448.7 | 224.35 | 12.32 | 0.00123 |
| GxE | 2 | 379.6 | 189.8 | 10.42 | 0.00238 |
| Res | 12 | 218.5 | 18.21 | | |

## Spikelets

His

| | Df | Sum Sq | Mean Sq | F value | Pr(>F) |
|---|---|---|---|---|---|
| T | 1 | 296.87 | 296.87 | 36.605 | 5.76E-05 |
| G | 2 | 235.36 | 117.68 | 14.511 | 0.000627 |
| GxE | 2 | 67.11 | 33.56 | 4.138 | 0.042984 |
| Res | 12 | 97.32 | 8.11 | | |

Gly

| | Df | Sum Sq | Mean Sq | F value | Pr(>F) |
|---|---|---|---|---|---|
| T | 1 | 14849 | 14849 | 48.892 | 1.45E-05 |
| G | 2 | 3249 | 1624 | 5.348 | 0.02184 |
| GxE | 2 | 4862 | 2431 | 8.004 | 0.00618 |
| Res | 12 | 3645 | 304 | | |

Ala

| | Df | Sum Sq | Mean Sq | F value | Pr(>F) |
|---|---|---|---|---|---|
| T | 1 | 1338 | 1338 | 2.804 | 0.12 |
| G | 2 | 37385 | 18693 | 39.169 | 5.49E-06 |
| GxE | 2 | 1059 | 529 | 1.109 | 0.361 |
| Res | 12 | 5727 | 477 | | |

Val

| | Df | Sum Sq | Mean Sq | F value | Pr(>F) |
|---|---|---|---|---|---|
| T | 1 | 115.5 | 115.5 | 6.058 | 0.02997 |
| G | 2 | 1149.4 | 574.7 | 30.135 | 2.10E-05 |
| GxE | 2 | 444.2 | 222.1 | 11.646 | 0.00155 |
| Res | 12 | 228.8 | 19.1 | | |

Pro

| | Df | Sum Sq | Mean Sq | F value | Pr(>F) |
|---|---|---|---|---|---|
| T | 1 | 285743 | 285743 | 64.986 | 3.48E-06 |
| G | 2 | 21558 | 10779 | 2.451 | 0.128 |
| GxE | 2 | 21318 | 10659 | 2.424 | 0.131 |
| Res | 12 | 52764 | 4397 | | |

Lys

| | Df | Sum Sq | Mean Sq | F value | Pr(>F) |
|---|---|---|---|---|---|
| T | 1 | 257.64 | 257.64 | 37.151 | 5.38E-05 |
| G | 2 | 121.45 | 60.72 | 8.756 | 0.004519 |
| GxE | 2 | 200.08 | 100.04 | 14.426 | 0.000642 |
| Res | 12 | 83.22 | 6.94 | | |

Asn

| | Df | Sum Sq | Mean Sq | F value | Pr(>F) |
|---|---|---|---|---|---|
| T | 1 | 1190836 | 1190836 | 50.366 | 1.25E-05 |
| G | 2 | 129755 | 64877 | 2.744 | 0.104 |
| GxE | 2 | 107997 | 53998 | 2.284 | 0.144 |
| Res | 12 | 283724 | 23644 | | |

Gln

| | Df | Sum Sq | Mean Sq | F value | Pr(>F) |
|---|---|---|---|---|---|
| T | 1 | 99488 | 99488 | 46.06 | 1.94E-05 |
| G | 2 | 95508 | 47754 | 22.11 | 9.45E-05 |
| GxE | 2 | 48063 | 24031 | 11.13 | 0.00185 |
| Res | 12 | 25917 | 2160 | | |

Asp

| | Df | Sum Sq | Mean Sq | F value | Pr(>F) |
|---|---|---|---|---|---|
| T | 1 | 3744 | 3744 | 11.241 | 0.005751 |
| G | 2 | 12092 | 6046 | 18.152 | 0.000235 |
| GxE | 2 | 1016 | 508 | 1.525 | 0.256927 |
| Res | 12 | 3997 | 333 | | |

Glu

| | Df | Sum Sq | Mean Sq | F value | Pr(>F) |
|---|---|---|---|---|---|
| T | 1 | 7581 | 7581 | 7.953 | 0.01546 |
| G | 2 | 49352 | 24676 | 25.887 | 4.44E-05 |
| GxE | 2 | 13657 | 6829 | 7.164 | 0.00897 |
| Res | 12 | 11439 | 953 | | |

Ser

| | Df | Sum Sq | Mean Sq | F value | Pr(>F) |
|---|---|---|---|---|---|
| T | 1 | 17323 | 17323 | 10.089 | 0.00798 |
| G | 2 | 27190 | 13595 | 7.918 | 0.00642 |
| GxE | 2 | 13493 | 6746 | 3.929 | 0.04869 |
| Res | 12 | 20605 | 1717 | | |

Thr

| | Df | Sum Sq | Mean Sq | F value | Pr(>F) |
|---|---|---|---|---|---|
| T | 1 | 503.5 | 503.5 | 10.769 | 0.006561 |
| G | 2 | 1870.5 | 935.3 | 20.003 | 0.000151 |
| GxE | 2 | 409 | 204.5 | 4.374 | 0.037429 |
| Res | 12 | 561.1 | 46.8 | | |

Leu

| | Df | Sum Sq | Mean Sq | F value | Pr(>F) |
|---|---|---|---|---|---|
| T | 1 | 0.85 | 0.845 | 0.145 | 0.7095 |
| G | 2 | 32.72 | 16.362 | 2.817 | 0.0993 . |
| GxE | 2 | 4.12 | 2.062 | 0.355 | 0.7083 |
| Res | 12 | 69.69 | 5.808 | | |

Ile

| | Df | Sum Sq | Mean Sq | F value | Pr(>F) |
|---|---|---|---|---|---|
| T | 1 | 8.41 | 8.41 | 3.149 | 0.101343 |
| G | 2 | 111.57 | 55.79 | 20.898 | 0.000123 |
| GxE | 2 | 138.65 | 69.33 | 25.97 | 4.37E-05 |
| Res | 12 | 32.03 | 2.67 | | |

Phe

| | Df | Sum Sq | Mean Sq | F value | Pr(>F) |
|---|---|---|---|---|---|
| T | 1 | 6.24 | 6.242 | 4.856 | 0.047831 |
| G | 2 | 52.5 | 26.251 | 20.42 | 0.000137 |
| GxE | 2 | 31.35 | 15.677 | 12.195 | 0.001286 |
| Res | 12 | 15.43 | 1.286 | | |

Gaba

| | Df | Sum Sq | Mean Sq | F value | Pr(>F) |
|---|---|---|---|---|---|
| T | 1 | 12715 | 12715 | 12.212 | 0.00443 |
| G | 2 | 11983 | 5991 | 5.754 | 0.01769 |
| GxE | 2 | 5506 | 2753 | 2.644 | 0.11184 |
| Res | 12 | 12494 | 1041 | | |

Total aa

| | Df | Sum Sq | Mean Sq | F value | Pr(>F) |
|---|---|---|---|---|---|
| T | 1 | 6065064 | 6065064 | 60.911 | 4.84E-06 |
| G | 2 | 2499274 | 1249637 | 12.55 | 0.00115 |
| GxE | 2 | 712552 | 356276 | 3.578 | 0.06043 |
| Res | 12 | 1194868 | 99572 | | |

Starch

| | Df | Sum Sq | Mean Sq | F value | Pr(>F) |
|---|---|---|---|---|---|
| T | 1 | 68882 | 68882 | 3.616 | 0.0815 |
| G | 2 | 330909 | 165454 | 8.686 | 0.00465 |
| GxE | 2 | 72498 | 36249 | 1.903 | 0.1915 |
| Res | 12 | 228588 | 19049 | | |

Glucose

| | Df | Sum Sq | Mean Sq | F value | Pr(>F) |
|---|---|---|---|---|---|
| T | 1 | 83.2 | 83.21 | 88.05 | 7.09E-07 |
| G | 2 | 349.7 | 174.84 | 185.01 | 9.61E-10 |
| GxE | 2 | 398.7 | 199.33 | 210.93 | 4.48E-10 |
| Res | 12 | 11.3 | 0.94 | | |

Fructose

| | Df | Sum Sq | Mean Sq | F value | Pr(>F) |
|---|---|---|---|---|---|
| T | 1 | 527 | 527 | 28.02 | 0.00019 |
| G | 2 | 1054.2 | 527.1 | 28.02 | 3.01E-05 |
| GxE | 2 | 604.7 | 302.3 | 16.07 | 0.000403 |
| Res | 12 | 225.7 | 18.8 | | |

Sucrose

| | Df | Sum Sq | Mean Sq | F value | Pr(>F) |
|---|---|---|---|---|---|
| T | 1 | 13.69 | 13.69 | 1.758 | 0.209529 |
| G | 2 | 273.61 | 136.81 | 17.566 | 0.000272 |
| GxE | 2 | 183.65 | 91.83 | 11.79 | 0.001472 |
| Res | 12 | 93.46 | 7.79 | | |

N

| | Df | Sum Sq | Mean Sq | F value | Pr(>F) |
|---|---|---|---|---|---|
| T | 1 | 0.02431 | 0.024315 | 3.548 | 0.0841 |
| G | 2 | 0.00911 | 0.004555 | 0.665 | 0.5324 |
| GxE | 2 | 0.04437 | 0.022185 | 3.237 | 0.0751 |
| Res | 12 | 0.08223 | 0.006853 | | |

C

| | Df | Sum Sq | Mean Sq | F value | Pr(>F) |
|---|---|---|---|---|---|
| T | 1 | 31.24 | 31.24 | 52.71 | 1.00E-05 |
| G | 2 | 6.848 | 3.424 | 5.777 | 0.0175 |
| GxE | 2 | 1.981 | 0.991 | 1.671 | 0.2289 |
| Res | 12 | 7.112 | 0.593 | | |

C/N

| | Df | Sum Sq | Mean Sq | F value | Pr(>F) |
|---|---|---|---|---|---|
| T | 1 | 0.34 | 0.34 | 0.04 | 0.8441 |
| G | 2 | 20.15 | 10.077 | 1.198 | 0.3355 |
| GxE | 2 | 53.29 | 26.644 | 3.167 | 0.0786 |
| Res | 12 | 100.94 | 8.412 | | |

**Supplementary Table 2: Tukey's HSD test outputs for the pairwise analysis of each metabolite in the 3 different genotypes in root, flag leaf and spikelets.** Vandana (V), WayRarem (WR) and 481-B. Each table contains the difference between the 2 sample means in the first column (diff), the lower (lwr) and upper (upr) bounds of the 95% confidence interval, and the adjusted probability for the difference test (adj p).

## Flag leaf

Ala

| | | | diff | lwr | upr | p adj |
|---|---|---|---|---|---|---|
| Drought | V | 481-B | -44.900 | -79.094 | -10.706 | 0.009 |
| Drought | WR | 481-B | -33.400 | -67.594 | 0.794 | 0.057 |
| Drought | WR | V | 11.500 | -22.694 | 45.694 | 0.860 |

Val

| | | | diff | lwr | upr | p adj |
|---|---|---|---|---|---|---|
| Drought | V | 481-B | -40.700 | -63.917 | -17.483 | 0.001 |
| Drought | WR | 481-B | -17.433 | -40.651 | 5.784 | 0.192 |
| Drought | WR | V | 23.267 | 0.049 | 46.484 | 0.049 |

Pro

| | | | diff | lwr | upr | p adj |
|---|---|---|---|---|---|---|
| Drought | V | 481-B | 353.033 | 158.165 | 547.901 | 0.001 |
| Drought | WR | 481-B | 604.000 | 409.132 | 798.868 | 0.000 |
| Drought | WR | V | 250.967 | 56.099 | 445.835 | 0.010 |

Lys

| | | | diff | lwr | upr | p adj |
|---|---|---|---|---|---|---|
| Drought | V | 481-B | -11.267 | -15.603 | -6.931 | 0.000 |
| Drought | WR | 481-B | -8.200 | -12.536 | -3.864 | 0.000 |
| Drought | WR | V | 3.067 | -1.269 | 7.403 | 0.238 |

Ser

| | | | diff | lwr | upr | p adj |
|---|---|---|---|---|---|---|
| Drought | V | 481-B | -86.867 | -123.760 | -49.973 | 0.000 |
| Drought | WR | 481-B | -53.800 | -90.694 | -16.906 | 0.004 |
| Drought | WR | V | 33.067 | -3.827 | 69.960 | 0.089 |

Thr

| | | | diff | lwr | upr | p adj |
|---|---|---|---|---|---|---|
| Drought | V | 481-B | -28.867 | -46.620 | -11.113 | 0.002 |
| Drought | WR | 481-B | -16.967 | -34.720 | 0.787 | 0.064 |
| Drought | WR | V | 11.900 | -5.854 | 29.654 | 0.284 |

Leu

| | | | diff | lwr | upr | p adj |
|---|---|---|---|---|---|---|
| Drought | V | 481-B | -16.967 | -26.010 | -7.923 | 0.000 |
| Drought | WR | 481-B | -1.867 | -10.910 | 7.177 | 0.979 |
| Drought | WR | V | 15.100 | 6.057 | 24.143 | 0.001 |

Gly

| | | | diff | lwr | upr | p adj |
|---|---|---|---|---|---|---|
| Drought | V | 481-B | -20.267 | -47.381 | 6.848 | 0.195 |
| Drought | WR | 481-B | 5.900 | -21.215 | 33.015 | 0.974 |
| Drought | WR | V | 26.167 | -0.948 | 53.281 | 0.061 |

Gln

| | | | diff | lwr | upr | p adj |
|---|---|---|---|---|---|---|
| Drought | V | 481-B | 39.900 | -81.673 | 161.473 | 0.871 |
| Drought | WR | 481-B | -8.133 | -129.706 | 113.440 | 1.000 |
| Drought | WR | V | -48.033 | -169.606 | 73.540 | 0.766 |

Asn

| | | | diff | lwr | upr | p adj |
|---|---|---|---|---|---|---|
| Drought | V | 481-B | 141.300 | -66.995 | 349.595 | 0.274 |
| Drought | WR | 481-B | -72.800 | -281.095 | 135.495 | 0.841 |
| Drought | WR | V | -214.100 | -422.395 | -5.805 | 0.043 |

Phe

| | | | diff | lwr | upr | p adj |
|---|---|---|---|---|---|---|
| Drought | V | 481-B | -19.800 | -39.228 | -0.372 | 0.045 |
| Drought | WR | 481-B | 1.567 | -17.862 | 20.995 | 1.000 |
| Drought | WR | V | 21.367 | 1.938 | 40.795 | 0.028 |

Total

| | | | diff | lwr | upr | p adj |
|---|---|---|---|---|---|---|
| Drought | V | 481-B | 346.100 | -267.899 | 960.099 | 0.450 |
| Drought | WR | 481-B | 267.733 | -346.266 | 881.732 | 0.691 |
| Drought | WR | V | -78.367 | -692.366 | 535.632 | 0.998 |

*Rice primary metabolites variation under drought*

Starch

| | | | diff | lwr | upr | p adj |
|---|---|---|---|---|---|---|
| Drought | V | 481-B | -1.653 | -3.321 | 0.014 | 0.052 |
| Drought | WR | 481-B | 1.503 | -0.164 | 3.171 | 0.087 |
| Drought | WR | V | 3.157 | 1.489 | 4.824 | 0.000 |

Glucose

| | | | diff | lwr | upr | p adj |
|---|---|---|---|---|---|---|
| Drought | V | 481-B | -4.467 | -12.790 | 3.856 | 0.499 |
| Drought | WR | 481-B | 2.333 | -5.990 | 10.656 | 0.928 |
| Drought | WR | V | 6.800 | -1.523 | 15.123 | 0.136 |

Fructose

| | | | diff | lwr | upr | p adj |
|---|---|---|---|---|---|---|
| Drought | V | 481-B | -12.067 | -33.715 | 9.582 | 0.461 |
| Drought | WR | 481-B | -1.200 | -22.848 | 20.448 | 1.000 |
| Drought | WR | V | 10.867 | -10.782 | 32.515 | 0.564 |

Sucrose

| | | | diff | lwr | upr | p adj |
|---|---|---|---|---|---|---|
| Drought | V | 481-B | -5.867 | -13.023 | 1.289 | 0.134 |
| Drought | WR | 481-B | -5.467 | -12.623 | 1.689 | 0.180 |
| Drought | WR | V | 0.400 | -6.756 | 7.556 | 1.000 |

# 1 Roots

His

| | | | diff | lwr | upr | p adj |
|---|---|---|---|---|---|---|
| Drought | V | 481-B | -28.300 | -33.595 | -23.005 | 0.000 |
| Drought | WR | 481-B | -34.600 | -39.895 | -29.305 | 0.000 |
| Drought | WR | V | -6.300 | -11.595 | -1.005 | 0.017 |

Gly

| | | | diff | lwr | upr | p adj |
|---|---|---|---|---|---|---|
| Drought | V | 481-B | -1.267 | -5.216 | 2.683 | 0.881 |
| Drought | WR | 481-B | -1.100 | -5.050 | 2.850 | 0.929 |
| Drought | WR | V | 0.167 | -3.783 | 4.116 | 1.000 |

Ser

| | | | diff | lwr | upr | p adj |
|---|---|---|---|---|---|---|
| Drought | V | 481-B | -163.400 | -296.757 | -30.043 | 0.014 |
| Drought | WR | 481-B | -157.467 | -290.824 | -24.110 | 0.018 |
| Drought | WR | V | 5.933 | -127.424 | 139.290 | 1.000 |

Thr

| | | | diff | lwr | upr | p adj |
|---|---|---|---|---|---|---|
| Drought | V | 481-B | -42.767 | -69.496 | -16.037 | 0.002 |
| Drought | WR | 481-B | -55.067 | -81.796 | -28.337 | 0.000 |
| Drought | WR | V | -12.300 | -39.029 | 14.429 | 0.645 |

Ala

| | | | diff | lwr | upr | p adj |
|---|---|---|---|---|---|---|
| Drought | V | 481-B | -77.433 | -133.757 | -21.109 | 0.006 |
| Drought | WR | 481-B | -88.100 | -144.424 | -31.776 | 0.002 |
| Drought | WR | V | -10.667 | -66.991 | 45.657 | 0.986 |

Val

| | | | diff | lwr | upr | p adj |
|---|---|---|---|---|---|---|
| Drought | V | 481-B | -34.567 | -57.783 | -11.351 | 0.003 |
| Drought | WR | 481-B | -21.500 | -44.716 | 1.716 | 0.076 |
| Drought | WR | V | 13.067 | -10.149 | 36.283 | 0.451 |

Leu

| | | | diff | lwr | upr | p adj |
|---|---|---|---|---|---|---|
| Drought | V | 481-B | -6.467 | -9.332 | -3.601 | 0.000 |
| Drought | WR | 481-B | -4.667 | -7.532 | -1.801 | 0.002 |
| Drought | WR | V | 1.800 | -1.065 | 4.665 | 0.344 |

Ile

| | | | diff | lwr | upr | p adj |
|---|---|---|---|---|---|---|
| Drought | V | 481-B | -9.067 | -14.851 | -3.282 | 0.002 |
| Drought | WR | 481-B | 0.267 | -5.518 | 6.051 | 1.000 |
| Drought | WR | V | 9.333 | 3.549 | 15.118 | 0.002 |

Pro

| | | | diff | lwr | upr | p adj |
|---|---|---|---|---|---|---|
| Drought | V | 481-B | -116.533 | -152.619 | -80.448 | 0.000 |
| Drought | WR | 481-B | -143.600 | -179.686 | -107.514 | 0.000 |
| Drought | WR | V | -27.067 | -63.152 | 9.019 | 0.193 |

Lys

| | | | diff | lwr | upr | p adj |
|---|---|---|---|---|---|---|
| Drought | V | 481-B | -10.833 | -15.913 | -5.754 | 0.000 |
| Drought | WR | 481-B | -24.100 | -29.180 | -19.020 | 0.000 |
| Drought | WR | V | -13.267 | -18.346 | -8.187 | 0.000 |

Asp

| | | | diff | lwr | upr | p adj |
|---|---|---|---|---|---|---|
| Drought | V | 481-B | -56.033 | -106.206 | -5.861 | 0.026 |
| Drought | WR | 481-B | -28.000 | -78.173 | 22.173 | 0.460 |
| Drought | WR | V | 28.033 | -22.139 | 78.206 | 0.459 |

Glu

| | | | diff | lwr | upr | p adj |
|---|---|---|---|---|---|---|
| Drought | V | 481-B | -56.600 | -126.011 | 12.811 | 0.138 |
| Drought | WR | 481-B | 4.367 | -65.044 | 73.777 | 1.000 |
| Drought | WR | V | 60.967 | -8.444 | 130.377 | 0.098 |

Gln

| | | | diff | lwr | upr | p adj |
|---|---|---|---|---|---|---|
| Drought | V | 481-B | -77.567 | -105.926 | -49.208 | 0.000 |
| Drought | WR | 481-B | -73.900 | -102.259 | -45.541 | 0.000 |
| Drought | WR | V | 3.667 | -24.692 | 32.026 | 0.998 |

Asn

| | | | diff | lwr | upr | p adj |
|---|---|---|---|---|---|---|
| Drought | V | 481-B | -272.000 | -572.839 | 28.839 | 0.085 |
| Drought | WR | 481-B | -394.267 | -695.106 | -93.428 | 0.009 |
| Drought | WR | V | -122.267 | -423.106 | 178.572 | 0.746 |

Gaba

| | | | diff | lwr | upr | p adj |
|---|---|---|---|---|---|---|
| Drought | V | 481-B | -21.433 | -48.346 | 5.480 | 0.152 |
| Drought | WR | 481-B | 1.200 | -25.713 | 28.113 | 1.000 |
| Drought | WR | V | 22.633 | -4.280 | 49.546 | 0.120 |

Phe

| | | | diff | lwr | upr | p adj |
|---|---|---|---|---|---|---|
| Drought | V | 481-B | -14.200 | -22.444 | -5.956 | 0.001 |
| Drought | WR | 481-B | -4.900 | -13.144 | 3.344 | 0.397 |
| Drought | WR | V | 9.300 | 1.056 | 17.544 | 0.024 |

Total aa

| | | | diff | lwr | upr | p adj |
|---|---|---|---|---|---|---|
| Drought | V | 481-B | -988.500 | -1470.095 | -506.905 | 0.000 |
| Drought | WR | 481-B | -1025.400 | -1506.995 | -543.805 | 0.000 |
| Drought | WR | V | -36.900 | -518.495 | 444.695 | 1.000 |

Starch

| | | | diff | lwr | upr | p adj |
|---|---|---|---|---|---|---|
| Drought | V | 481-B | 0.137 | -2.455 | 2.728 | 1.000 |
| Drought | WR | 481-B | 3.457 | 0.865 | 6.048 | 0.008 |
| Drought | WR | V | 3.320 | 0.729 | 5.911 | 0.010 |

Glucose

| | | | diff | lwr | upr | p adj |
|---|---|---|---|---|---|---|
| Drought | V | 481-B | -4.067 | -4.929 | -3.204 | 0.000 |
| Drought | WR | 481-B | -2.000 | -2.862 | -1.138 | 0.000 |
| Drought | WR | V | 2.067 | 1.204 | 2.929 | 0.000 |

Fructose

| | | | diff | lwr | upr | p adj |
|---|---|---|---|---|---|---|
| Drought | V | 481-B | -10.067 | -14.141 | -5.992 | 0.000 |
| Drought | WR | 481-B | -6.400 | -10.475 | -2.325 | 0.002 |
| Drought | WR | V | 3.667 | -0.408 | 7.741 | 0.087 |

Sucrose

| | | | diff | lwr | upr | p adj |
|---|---|---|---|---|---|---|
| Drought | V | 481-B | -6.267 | -9.608 | -2.925 | 0.000 |
| Drought | WR | 481-B | -6.767 | -10.108 | -3.425 | 0.000 |
| Drought | WR | V | -0.500 | -3.841 | 2.841 | 0.995 |

N

| | | | diff | lwr | upr | p adj |
|---|---|---|---|---|---|---|
| Drought | V | 481-B | -0.082 | -0.381 | 0.216 | 0.932 |
| Drought | WR | 481-B | 0.095 | -0.204 | 0.394 | 0.884 |
| Drought | WR | V | 0.177 | -0.121 | 0.476 | 0.398 |

C

| | | | diff | lwr | upr | p adj |
|---|---|---|---|---|---|---|
| Drought | V | 481-B | -5.386 | -7.339 | -3.432 | 0.000 |
| Drought | WR | 481-B | 2.334 | 0.380 | 4.287 | 0.017 |
| Drought | WR | V | 7.720 | 5.766 | 9.673 | 0.000 |

C/N

| | | | diff | lwr | upr | p adj |
|---|---|---|---|---|---|---|
| Drought | V | 481-B | -2.074 | -13.778 | 9.630 | 0.989 |
| Drought | WR | 481-B | -0.663 | -12.367 | 11.041 | 1.000 |
| Drought | WR | V | 1.411 | -10.293 | 13.115 | 0.998 |

## Spikelets

His

| | | | diff | lwr | upr | p adj |
|---|---|---|---|---|---|---|
| Drought | V | 481-B | 13.067 | 5.256 | 20.877 | 0.001 |
| Drought | WR | 481-B | 3.433 | -4.377 | 11.244 | 0.684 |
| Drought | WR | V | -9.633 | -17.444 | -1.823 | 0.013 |

Gly

| | | | diff | lwr | upr | p adj |
|---|---|---|---|---|---|---|
| Drought | V | 481-B | 11.567 | -36.229 | 59.363 | 0.960 |
| Drought | WR | 481-B | -56.367 | -104.163 | -8.571 | 0.018 |
| Drought | WR | V | -67.933 | -115.729 | -20.137 | 0.005 |

Gln

| | | | diff | lwr | upr | p adj |
|---|---|---|---|---|---|---|
| Drought | V | 481-B | 144.400 | 16.946 | 271.854 | 0.024 |
| Drought | WR | 481-B | -138.800 | -266.254 | -11.346 | 0.030 |
| Drought | WR | V | -283.200 | -410.654 | -155.746 | 0.000 |

Glu

| | | | diff | lwr | upr | p adj |
|---|---|---|---|---|---|---|
| Drought | V | 481-B | -87.733 | -172.407 | -3.059 | 0.041 |
| Drought | WR | 481-B | -177.800 | -262.474 | -93.126 | 0.000 |
| Drought | WR | V | -90.067 | -174.741 | -5.393 | 0.035 |

Ser

| | | | diff | lwr | upr | p adj |
|---|---|---|---|---|---|---|
| Drought | V | 481-B | -7.833 | -121.478 | 105.811 | 1.000 |
| Drought | WR | 481-B | -143.600 | -257.244 | -29.956 | 0.011 |
| Drought | WR | V | -135.767 | -249.411 | -22.122 | 0.017 |

Thr

| | | | diff | lwr | upr | p adj |
|---|---|---|---|---|---|---|
| Drought | V | 481-B | -4.300 | -23.053 | 14.453 | 0.968 |
| Drought | WR | 481-B | -30.400 | -49.153 | -11.647 | 0.002 |
| Drought | WR | V | -26.100 | -44.853 | -7.347 | 0.005 |

Val

| | | | diff | lwr | upr | p adj |
|---|---|---|---|---|---|---|
| Drought | V | 481-B | 2.967 | -9.010 | 14.943 | 0.956 |
| Drought | WR | 481-B | -6.133 | -18.110 | 5.843 | 0.545 |
| Drought | WR | V | -9.100 | -21.076 | 2.876 | 0.184 |

Lys

| | | | diff | lwr | upr | p adj |
|---|---|---|---|---|---|---|
| Drought | V | 481-B | 3.400 | -3.822 | 10.622 | 0.624 |
| Drought | WR | 481-B | -10.533 | -17.756 | -3.311 | 0.004 |
| Drought | WR | V | -13.933 | -21.156 | -6.711 | 0.000 |

Ile

| | | | diff | lwr | upr | p adj |
|---|---|---|---|---|---|---|
| Drought | V | 481-B | -1.567 | -6.048 | 2.914 | 0.841 |
| Drought | WR | 481-B | -1.833 | -6.314 | 2.648 | 0.741 |
| Drought | WR | V | -0.267 | -4.748 | 4.214 | 1.000 |

Phe

| | | | diff | lwr | upr | p adj |
|---|---|---|---|---|---|---|
| Drought | V | 481-B | 1.100 | -2.010 | 4.210 | 0.834 |
| Drought | WR | 481-B | 1.400 | -1.710 | 4.510 | 0.664 |
| Drought | WR | V | 0.300 | -2.810 | 3.410 | 0.999 |

Total aa

| | | | diff | lwr | upr | p adj |
|---|---|---|---|---|---|---|
| Drought | V | 481-B | 583.833 | -281.580 | 1449.247 | 0.278 |
| Drought | WR | 481-B | -799.300 | -1664.713 | 66.113 | 0.077 |
| Drought | WR | V | -1383.133 | -2248.547 | -517.720 | 0.002 |

Starch

| | | | diff | lwr | upr | p adj |
|---|---|---|---|---|---|---|
| Drought | V | 481-B | -468.933 | -847.454 | -90.412 | 0.013 |
| Drought | WR | 481-B | -137.300 | -515.821 | 241.221 | 0.820 |
| Drought | WR | V | 331.633 | -46.888 | 710.154 | 0.099 |

Glucose

| | | | diff | lwr | upr | p adj |
|---|---|---|---|---|---|---|
| Drought | V | 481-B | 16.833 | 14.167 | 19.499 | 0.000 |
| Drought | WR | 481-B | -3.667 | -6.333 | -1.001 | 0.006 |
| Drought | WR | V | -20.500 | -23.166 | -17.834 | 0.000 |

Fructose

| | | | diff | lwr | upr | p adj |
|---|---|---|---|---|---|---|
| Drought | V | 481-B | 19.933 | 8.039 | 31.827 | 0.001 |
| Drought | WR | 481-B | -12.533 | -24.427 | -0.639 | 0.037 |
| Drought | WR | V | -32.467 | -44.361 | -20.573 | 0.000 |

Sucrose

| | | | diff | lwr | upr | p adj |
|---|---|---|---|---|---|---|
| Drought | V | 481-B | -5.900 | -13.554 | 1.754 | 0.173 |
| Drought | WR | 481-B | -16.633 | -24.287 | -8.980 | 0.000 |
| Drought | WR | V | -10.733 | -18.387 | -3.080 | 0.005 |

N

| | | | diff | lwr | upr | p adj |
|---|---|---|---|---|---|---|
| Drought | V | 481-B | -0.065 | -0.292 | 0.162 | 0.920 |
| Drought | WR | 481-B | -0.002 | -0.229 | 0.225 | 1.000 |
| Drought | WR | V | 0.064 | -0.163 | 0.291 | 0.927 |

C/N

| | | | diff | lwr | upr | p adj |
|---|---|---|---|---|---|---|
| Drought | V | 481-B | 1.402 | -6.552 | 9.356 | 0.990 |
| Drought | WR | 481-B | -0.076 | -8.031 | 7.878 | 1.000 |
| Drought | WR | V | -1.479 | -9.433 | 6.476 | 0.987 |

**Supplementary Table 3:** List of primers used for qRT-PCR.

| Locus ID | Gene Name | Primer Name | Primer Sequence (5'-3') |
|---|---|---|---|
| Os08g01770 | adenylate kinase | 08AK_CF3 | CCCAAGAAGCTATTCGCAAG |
| | | 08AK_CR3 | ATCAAGCCGTCTCCCAACTA |
| Os07g42490 | sucrose synthase | 07SucroSy_CF2 | CTACAGCCCCACCAGATCAT |
| | | 07SucroSy_CR2 | AGGGACACTCAGCTCCTCAA |
| Os01g71990 | pyrroline-5-carboxylate reductase | 1P5CR_CF2 | ATCTCTGTGAAGCCGCAGAT |
| | | 1P5CR_CR2 | CAACAGCTGAAGGGGTGTTT |
| Os05g38150 | pyrroline-5-carboxylate synthase | 5P5CS_CF2 | CAGTTTAGCAGGACTGTTGG |
| | | 5P5CS_CR2 | TGTCATGCCTCCTCTACCTA |
| Os04g55720 | D-3-phosphoglycerate dehydrogenase | 04PGDH_CF2 | ATCTTGTTGGATGGCTCACC |
| | | 04PGDH_CR2 | CAAGCTCACATCAACCTGGA |
| Os01g64630 | actin | 01Act qCF1 | ACTCCCGCATGCTATTCTTCGAC |
| | | 01Act qCR1 | TGAAGGAGTAACCCCTCTCTGTGA |
| Os07g38730 | tubulin | 07Tub qCF2 | CCTCTGTTGTCCCTGGTGGTGA |
| | | 07Tub qCR2 | GGTCGATGCGCGAGAAAACCTC |
| Os07g01760 | glutamate glyoxylate aminotransferase | 07AmTrans qCF2 | ACCCGCTTTATTCTGCTGCCATTT |
| | | 07AmTrans qCR2 | CGAAGTCAAGTCCCCAGTTAGCCT |
| Os08g39300 | alanine-glyoxylate aminotransferase | 08AmTrans qCF1 | TCTACAAGATGGGCACCTACTGGC |
| | | 08AmTrans qCR1 | CTTCCTCGAAGATGAGGTCGAGGG |
| Os07g46460 | Fd-GOGAT | 07FGDS qCF1 | CAATATTGTCCGGGATGCCCTTGT |
| | | 07FGDS qCR1 | TGAACCGTCACCGGAGTCATTGTC |
| Os02g50240 | glutamine synthase | 02GluSyn qCF2 | ATTGAGCAAGAGTACACCCTCC |
| | | 02GluSyn qCR2 | TAGTAAGGACCCTGAGGACCAG |
| Os08g25734 | gluc-1-phosphate adenylyltransferase | 08G1PAT qCF1 | CTTGACGACGTGAGGGCAAAAGAA |
| | | 08G1PAT qCR1 | TCACGGAGGAGCTGAAGCATTACA |
| Os02g02890 | cyclophilin 2 | OsCyclo_qCF1 | GTGGTGTTAGTCTTTTTATGAGTTCGT |
| | | OsCyclo_qCR1 | ACCAAACCATGGGCGATCT |
